# Supplementary material for: Effects of Square-Stepping Exercise on cognitive function in early geriatric rehabilitation: A randomized controlled explorative study
Source: PLoS One. 2026 Jan 5;21(1):e0338695. doi: 10.1371/journal.pone.0338695 (PMC12768348; doi:10.1371/journal.pone.0338695)
Supplement: S1 Appendix — (DOCX) [file pone.0338695.s001.docx]

Akademisches Lehrkrankenhaus

der Universität Göttingen

Medizinische Klinik III
Geriatrie

**Dr. med. Helmut Ackermann**

Chefarzt

Innere Medizin- Geriatrie-

Nephrologie

Telefon: (0471) 299-3690

Telefax: (0471) 299-3743

Internet: www.klinikum-bremerhaven.de

Klinikum Bremerhaven, Postbrookstr. 103, 27574 Bremerhaven

**Step by Step: A randomised controlled pilot study on the effect of ‘Square Stepping Exercise’ on cognitive functioning and mobility of inpatients in an acute geriatric rehabilitation unit**

**Study protocol**

1. **Title**

Step by Step: A randomised controlled pilot study on the effect of ‘Square Stepping Exercise’ on cognitive functioning and mobility of inpatients in an acute geriatric rehabilitation unit

1. **Introduction**

Mobility in older people is significantly influenced by walking ability and cognitive performance. Both aspects are important determinants of physical health and psychological well-being. It is well established that walking requires the interaction between a number of higher cognitive functions, that the cognitive demands of walking increase with age and that simultaneously cognitive performance decreases with age. This interplay between physical activity and cognitive performance in old age has been studied several times within the framework of the dual-task paradigm and clear interferences between the two have been demonstrated.

A decrease in walking ability and gait safety is associated with multiple limitations and a decrease in overall independence. Long-term studies have also shown that clinical changes in gait are often associated with higher risk of developing dementia.

There are already many clinical studies which examine the effects of improved mobility through physical activity. The umbrella term of physical activity includes sports as well as targeted training to improve individual functions. Studies on physical activity often focus on the increase in functional fitness of the lower extremities for fall prevention. Shigematsu and Okura developed the "Square Stepping Exercise" (SSE) which they tested and published first in 2006 as a targeted training with seniors living independently. The exercise is practised on a mat divided into 40 squares and is based on the execution of different step patterns with lunges to the front, back and side with increasing difficulty. The authors aimed to both reduce the risk of falls and improve the functional fitness of the lower extremities through training. According to the principle of proactive and reactive reaction improvement, the training is intended to optimise the execution of a corrective step to regain balance as a result of stumbling, in order to prevent falls. By activating agonists and antagonists of the lower extremities fitness should also be improved.

There are now numerous studies on the influence of physical activity on cognitive abilities in older people. These have shown significant effects on executive and memory functions. In particular, forms of exercise such as walking, aerobics and endurance training have been investigated. However, these studies also show that targeted physical activity only has a significant effect on cognitive performance when both physical and cognitive performance demands are made during training.

Because SSE combines both physical and cognitive activities the dual task requirement is particularly high in SSE training. The subjects must first memorise the trainer's instruction in order to be able to perform the patterns correctly and then implement the instruction motorially. Since the training makes demands on motor implementation as well as on cognitive multi tasking and working memory, it is postulated that SSE training can have a positive effect on overall cognitive performance.

To date SSE has been studied exclusively on independently living seniors. This prompted us to investigate the effectiveness of SSE in the context of inpatient acute rehabilitation in comparison to conventional physiotherapy. In addition to exercises to strengthen the lower extremities conventional physiotherapy usually includes gait training and other exercises to maintain activities relevant to everyday life, such as climbing stairs.

We have been using elements of SSE in our geriatric acute rehabilitation for several years, but so far without formal standardisation. There have been no studies within the setting of inpatient geriatric acute rehabilitation so far, which is why this study is intended as a pilot project to examine SSE training in comparison to conventional physiotherapy for an improvement in gait parameters and executive cognitive functions.

1. **Responsibilities**

Director of studies

Dr. Svenja Tietgen

Department of Geriatrics

Medical Director Dr. med. Helmut Ackermann, Consultant Geriatrican

Clinical Center Bremerhaven Reinkenheide gGmbH

Postbrookstraße 103

27574 Bremerhaven

Further staff member

Katja Fränzel

Department of Geriatrics

Medical Director Dr. med. Helmut Ackermann, Consultant Geriatrican

Clinical Center Bremerhaven Reinkenheide gGmbH

Postbrookstraße 103

27574 Bremerhaven

Participating institutions/collaborating partners

PD Dr. Ellen Freiberger

Institute for Biomedicine of Aging FAU Erlangen-Nürnberg

Kobergerstr. 60

90408 Nürnberg

Professor Ryosuke SHIGEMATSU, Ph.D.

School of Health and Sport Science,

Chukyo University

101 Tokodachi, Toyota, Aichi,

Japan 470-0393

Prof. Dr. Tania Zieschang

Department of Geriatrics

Carl von Ossietzky University Oldenburg

Faculty VI Medicine and Health Sciences

Department of Health Services Research

Ammerländer Heerstraße 140, Building V04, Room 118

26129 Oldenburg

Dr. Jessica Koschate

Department of Geriatrics

Carl von Ossietzky University Oldenburg

Faculty VI Medicine and Health Sciences

Department of Health Services Research

Ammerländer Heerstraße 140, Building V04, Room 118

26129 Oldenburg

External funding

None. No third-party funds have been applied for.

1. **Background**

Mobility in older age is closely associated with physical and mental health (Yeom, Fleury & Keller, 2008; Webber, Porter & Menec, 2010). Webber & colleagues (2010) attempted to operationalise the concept of mobility in old age and summarised five basic categories of determining factors: Cognitive, psychosocial, physical, environmental and financial determinants. A crucial aspect of the physical determinant is the ability to walk. Cognitive ability is also crucial for the largely independent performance of activities of daily living. Both variables are important for maintaining independence, which in turn promotes social participation and increases the quality of life of older people.

Similarly, the 2015 World Health Organisation (WHO) report on ageing and health focuses on functionality as a crucial factor for healthy ageing. The concept of "Healthy Aging" is defined as the process of developing and maintaining functional capacity with the goal of individual well-being. The intrinsic capacity of a person is considered to be the decisive factor of this functional capacity, which is composed of physical and likewise mental abilities. It is assumed that the assessment of functional capacity is a better predictor of a positive outcome in old age than individual diseases or the extent of multimorbidity. The high significance of functionality, which is based on both physical and mental performance and their interaction, can be derived from this.

Especially for the early detection of dementia and fall risks, research on gait changes in old age has attracted increasing interest in recent years. Gait and cognitive parameters are no longer understood as two independent processes, but rather as an interaction between both within a mutually dependent relationship (Holtzer, Verghese, Xue & Lipton, 2006 Al-Yahya et al. 2011; Montero-Odasso et al. 2012, Cohen, Verghese & Zwerling, 2016). The previously widespread hypothesis that walking is a highly overlearned, automated rhythmic movement requiring little cognitive input has recently been refuted (Hausdorff and colleagues (2005)). Their study shows that walking requires the interaction of higher cognitive functions such as perception, action planning, attention and memory processes. The authors deduce that even routine walking represents a complex cognitive task, an insight which opens new approaches for the treatment of gait abnormalities and fall risk in older people. With age, attentional demands on gait and balance control change, increasing the cognitive demands of walking in old age (Woollacott & Shumway-Cook, 2002¸Hausdorff et al. 2005).

In the course of a normal ageing process, structural and functional changes also occur at the brain level. A modification of these results from physiological degradation processes, but also from restructuring processes of the ageing brain. It is now assumed that there is an interaction between the structural changes caused by natural ageing processes and changing cognitive demands on the brain in old age (Brand & Markowitsch 2004). Ageing-related degradation processes show up more strongly in some structures at an earlier point in time, and less strongly or not at all in others. In particular, the prefrontal and medial temporal structures show the clearest age effects. As a neuropsychological correlate, this manifests itself in a reduction of executive and mnestic functions (Hedden & Yoon, 2006; Morrison & Baxter, 2012).

Electrophysiological investigations were able to demonstrate altered neuronal activity in older subjects compared to younger subjects, suggesting neuronal restructuring in old age. It could be shown that older subjects use different neuronal loops to cope with test requirements. In particular, stronger prefrontal activation could be demonstrated (Cabeza et al. 2002; Cabeza et al. 2004). Current research on altered neuronal activity in old age focuses on cognitive reserve capacity, which is associated with the structures of the prefrontal cortex among other things. A recent longitudinal study by Soshi and colleagues (2021) suggests that even short-term cognitive training interventions over three months can induce prefrontal plasticity related to cognitive performance in older adults.

Recent research has increasingly focused on the neural mechanisms underlying gait control in older people. Harada, Miyai, Suzuki and Kobota (2009) used functional near-infrared spectroscopy (fNIRS) to investigate which cortical activation patterns are associated with gait speed in relation to walking ability in older people. The results suggest that gait speed is controlled by the left prefrontal cortex, the supplementary motor cortex and the sensorimotor cortex. Increased prefrontal activation during cognitive demands, including spatial and verbal working memory is associated with older age (Reuter-Lorenz et al, 2000). The meta-analysis on fNIRS studies on the changes in brain activity during walking in adults with and without neurological diseases by Bishnoi, Holtzer and Hernandez (2021) confirms the assumption of a significant increase in brain activation in the prefrontal cortex during walking under dual-task conditions compared to standing and simple walking. At the same time, it is assumed that a stronger left-dominant prefrontal activation is associated with an age-related loss of cognitive performance (Logan et al., 2002). The age-related decline in gait capacity with concomitant decline in executive functions involving the prefrontal cortex highlights the interaction between gait and executive performance. A systematic review by Kearney et al (2013) shows that age-related changes in executive functions and information processing speed affect walking ability and the risk of falls in older people (Kearney et al. 2013). Based on the assumption that cognitive performance capacity becomes increasingly limited in older age, the dual-task paradigm has been used in clinical studies to detect interference between walking and cognitive functions. Holtzer, Ross and Izzetoglu (2020) showed a positive correlation of variability in neuronal activity in the prefrontal cortex and gait performance using fNIRS during active walking in older adults which could only be mapped under dual-task conditions.

Test performance in dual-task tasks serves to quantify executive functions which are considered to be decisive for cognitive reserve capacity (Herman et al. 2010) and are associated with increased activity in the dorsolateral prefrontal cortex according to current imaging studies (Herman et al, 2010; Szameitat, 2002). Studies with dual-task requirements show an interference of gait speed and executive functions (Smith, Cusack & Blake, 2016). In their meta-analysis on cognitive motor interference during walking, Al-Yahya et al. (2011) showed a significant effect of different cognitive tasks on gait speed. The meta-regression analysis indicated a strong correlation between increasing age and the reduction in walking speed, as well as between cognitive performance and walking speed under dual-task conditions. The extent of interference can go as far as interruption of the motor task in the presence of increased cognitive demand. As the "stops walking when talking" phenomenon, this maximum interference has become known as a predictor of falls in the inpatient geriatric setting (Lundin-Olsson, Nyberg & Gustafson 1997).

The effect of physical training on cognitive performance and physical function parameters, such as strength, mobility, gait and balance, in older people has already been discussed in several systematic reviews. These consistently indicate that multi-component training, including strength, aerobic, balance and flexibility training seem to be the most suitable for improving physical function parameters. There is no consensus yet on which training features, in terms of type, duration, intensity, frequency and combination, are most effective in the long term. In a cross-sectional study, Falck et al. (2017) investigated the relationship between physical activity and the development of Mild Cognitive Impairment (MCI) and concluded that >150min/week of physical activity is associated with better cognitive abilities. Conversely, it needs to be investigated whether MCI leads to neurobiological changes that result in a reduction of physical activity. Oswald and colleagues (2006) investigated the long-term effect of cognitive and physical training in independently living seniors aged 75 to 93 years in a longitudinal study over 5 years. Significant training effects after 5 years compared to baseline could be measured for the group of subjects who received combined cognitive and physical training. The recent Cochrane Review by Jadczak et al. (2018) included seven systematic reviews examining the effectiveness of physical training on physical functioning in frail older people. The review concludes that multicomponent training including resistance, aerobic, balance and flexibility exercises, can be recommended for frail older adults to increase muscle strength, gait speed, balance and overall physical performance.

It is now considered certain that regular cognitive stimulation has a positive effect on cognitive performance in old age (Ball et al. 2002; Willis et al. 2006). However, a heterogeneous picture emerges in the quantification of cognitive abilities. Colombe & Kramer (2003), for example, investigated the effect of aerobic training on cognitive performance in seniors in a meta-analysis. A total of 18 studies published between 1966 and 2001 were included. Regardless of the type of cognitive task on which the respective studies were based and regardless of the personal characteristics of the subjects cognitive performance was increased by an average of 0.5 standard deviations by the fitness training applied. The authors showed that the training effects were robust but of benefit only for selective cognitive function domains. The analysis revealed the largest process-specific benefit of physical fitness training on executive and cognitive control processes. Colombe and Kramer concluded that those cognitive functional domains for which an age-associated decline in performance has been demonstrated in previous studies can be improved by aerobic training.

The meta-analysis of the effect of physical activity on physical and cognitive functions in older adults by Falck et al. (2019) confirms the significant effect of physical training on both physical and cognitive functions postulated in the previous studies.

The Square Stepping Exercise involves a high dual task demand, which is why it is postulated that the training improves cognitive performance (Teixeira et al. 2013). Originally developed by Shigematsu and Okura in Japan, the primary aim of the programme was to improve functional fitness of the lower extremities in order to improve the walking ability of older people in the general population and to minimise the risk of falls through higher functional fitness (Shigematsu & Okura, 2006; Shigematsu et al. 2008b).

SSE is practised on a thin mat divided into 40 squares. Step patterns, depending on the level of difficulty, are walked with lunges to the front, back and side. The patterns can also be run in toe or heel gait. Within the training, the complexity of the walking patterns increases. Following the principle of proactive and reactive reaction improvement, the running patterns serve to improve the restoration of balance after a stumble through a corrective step.

**Rationale for the proposed study**

According to the "Healty Aging" concept of the WHO (2015), training to promote and maintain mobility in older people should target both physical and cognitive function.

Fisseha et al (2017) provided an overview of the study landscape in their systematic review and meta-analysis of the effectiveness of SSE on fall prevention and fall injuries. SSE was found to be effective in reducing fear of falling in older persons as well as improving their subjectively perceived health status. Due to a paucity of evidence there is no certainty so far whether SSE is superior in comparison to other interventions in reducing falls and fall injuries.

Our literature search on the impact of SSE on cognitive performance revealed two clinical studies focusing on cognition. Teixeira et al (2013) were the first to investigate the impact of 16 weeks of SSE training on cognitive performance in older people. The authors concluded that SSE, as a form of global cognitive stimulation, has a positive impact on cognitive performance, particularly in focused attention performance as well as cognitive flexibility in older people. Shigematsu et al (2014) investigated the effect of daily SSE training in healthy seniors in a home setting compared to daily SSE training every 14 days over a period of six months on performance in 5 cognitive tests of executive and memory functions. In one test procedure on executive functions a significant improvement in test performance was achieved with continuous SSE training. Memory parameters were improved in both groups. However, the quantification of the cognitive parameters in the pilot study has a rather global character, which could explain the small effect size in the other three test procedures.

Studies to date suggest that regular use of SSE training provides both cognitive stimulation and physical training, which can simultaneously improve walking ability and cognitive performance. SSE is simple and economical to perform, as it only requires a flat training mat. It is less physically demanding than aerobic, strength or fitness training and can be practised in the home environment. Thus SSE offers an alternative to outdoor training in bad weather conditions, an alternative to external sports for less mobile seniors and an alternative to physically demanding activity for seniors in a reduced general condition.

In the meantime feasibility studies have been conducted occasionally on the use of SSE to improve clinical symptoms in various disease conditions, such as multiple sclerosis (Sebastião et al. 2018) and diabetes (Shellington et al. 2018). The results supported the feasibility and acceptability of training. There was also evidence of a possible improvement in executive functions in adults with type 2 diabetes.

A double-blind RCT is currently underway in Mexico to investigate the effect of SSE training in 60-65 year olds on vascular and cognitive function (Sanchez-Arenas 2020), with results pending.

So far studies examining interventions designed o reduce the risk of falls or to increase the cognitive and physical function of older people training has targeted independently living seniors in a home environment with the study participants having been recruited via the media. So far very little emphasis has been placed on the development of interventions which focus on the improvement of walking abilities and cognitive performance in inpatient geriatric settings. Furthermore little research has been completed on the interaction of cognitive training and physical training in geriatric patients. Inpatient geriatric rehabilitation is based on the overarching rehabilitation goal of improving mobility, which includes improving social interaction and avoiding or reducing dependence on caregivers to maintain independence (Swoboda & Sieber, 2010). Within the framework of multidisciplinary treatment in the geriatric team the physical mobilisation of patients is mainly carried out by physiotherapeutic treatment. In addition to application procedures such as manual therapies, physiotherapeutic pain therapy and lymphatic drainage, functional training is used to increase strength, endurance, balance, stance and gait security, which, however, has not yet been subject to standardisation.

Due to the high dual-task demands of SSE and the presumed effectiveness of training in improving functional fitness of the lower limbs (Shigematsu & Okura, 2006; Shigematsu et al. 2008; Shigematsu et al. 2008b) an effect on walking ability and cognitive performance in the functional area of executive functions and working memory is assumed.

By applying a uniform SSE training plan to the patients typical for an inpatient geriatric early rehabilitation environment our pilot study "Step by Step" should increase our knowledge about the interactions between ageing, exercise, cognitive functioning and mobility significantly. Previous studies on the effect of SSE were conducted on a group of independently living seniors whereas our research setting will be conducted on a broadly diversified patient group which is highly representative of the average ageing population in general. Our research will also avoid recruitment bias which has been a problem in previously conducted studies where subjects were recruited through media. This recruitment method is likely to favour subjects with higher levels of cognitive and physical fitness and greater mobility, and with a higher pre-existing motivation to increase physical performance. Seniors who have less access to media or who have a lower level of mobility are less likely to be reached through this recruitment route.

So far, the effect of SSE training on cognitive performance has mostly been investigated on the basis of a global approach. Screening instruments such as the Mini Mental Status Test, the Montreal Cognitive Assessment or subtests from various test batteries were frequently used. Those tools are comparatively crude and unsophisticated. Specific measurement of individual cognitive functional areas is not possible with such instruments. Screening instruments used previously are not usually age-, education- or gender-adjusted and are more susceptible to daily fluctuations and other influencing factors. Statistically significant changes would escape detection with such tools. In this context the fact that the concept of executive functioning is a heterogeneous construct also poses a particular challenge. The results from clinical studies on the reciprocal effect of physical activity and executive functions are not easily comparable due to the consideration of different domains that the authors assign to executive functions. In this pilot study, a dedicated and standardised measurement of different subdomains of the executive functions will therefore be carried out in order to be able to specifically assign the effect of the dual-task requirement from the SSE training to individual subdomains and to uncover possible correlations between these. With the aim of increasing the success of rehabilitation in inpatient geriatric early rehabilitation, these findings can finally be implemented in therapeutic practice.

In the pilot study "Step by Step", SSE training in the context of inpatient geriatric early rehabilitation is thus to be compared with conventional rehabilitative physiotherapy. The primary aim of this study is to investigate the possibility of implementing SSE training in the inpatient geriatric setting. Furthermore, the implementation of the study could provide information on whether a significant improvement on cognitive and gait parameters can be measured after three weeks of SSE training. The quantification of the cognitive parameters is carried out in a dedicated and standardised way for individual functional areas of the executive functions, in order to better represent and understand specific effects of the SSE training. The implementation of this pilot study could thus provide information on whether the implementation of SSE training in inpatient geriatric physiotherapeutic treatment can achieve greater rehabilitation success with regard to mobility for the patients. This could contribute to an optimisation of the treatment concept and would open up new starting points for research.

1. **Objectives/Outcome**

Research question 1: Can a change in lower limb physical function be measured with a combination treatment of SSE and conventional physical therapy compared to the control group receiving only conventional physical therapy?

Research question 2: Can a combination treatment of SSE and usual physiotherapy achieve an increase in executive cognitive performance compared to usual physiotherapy only?

Research question 3: Does a combination of SSE and conventional physical therapy increase mobility more than conventional physical therapy only?

Research question 4: Does subjective quality of life improve with a combination treatment of SSE and usual physiotherapy compared to usual physiotherapy only?

Research question 5: Does a combination treatment of SSE and usual physiotherapy reduce fear of falling compared with usual physiotherapy only?

1. **Target figures**

Physical parameters

- Step length (cm)
- Track width (cm, additionally with variance)
- Cadence (steps/min)
- Walking speed / gait speed (m/s)
- Timed "Up & Go" test
- Short Physical Performance Battery (SPPB)

Cognitive parameters

- Basal attention function / simple reaction processing speed
- Divided attention performance
- Cognitive conversion ability
- Working memory
- Memory span

Fear of falling

- Reduction of Fear of Falling Questionnaire (FES-I)

Subjective quality of life

- Questionnaire (EuroQuol)

1. **Trial Design**

Monocentric

The study will be conducted exclusively in the geriatric department of the Klinikum Bremerhaven- Reinkenheide.

Two-arm study

The study consists of an intervention and a control group.

Randomisation

There is simple randomisation.

Blinding

There is a simple blinding. The subjects know which treatment they are receiving, the investigators do not.

1. **Study Population (collective)**

Study population

Patients in geriatric complex treatment at KBR

Inclusion and exclusion criteria

Inclusion criteria

- Ability to walk short distances (10m) without aids in company
- Capacity to consent exists (MMSE > 22 pts)
- Sufficient cognitive skills to implement the training programme
- Participation in inpatient geriatric early rehabilitation
- Sufficient knowledge of German or English

Exclusion criteria

- Aphasia to an extent that participation in the study is not possible
- Severe visual limitations
- High grade presbycusis
- Severe impairment of physical functionality and limitations of functions of the arms and legs with the inability to walk

Number of study participants: 60 participants in total

- n= 30 in the intervention group
- n= 30 in the control group

Recruitment channels and measures

During the admission of the patients to the geriatric early rehabilitation clinic, they are checked for fulfilment of the inclusion and exclusion criteria and included after being informed about the study and signing the consent form.

Recruitment period: 18 months

After a one-month caseload planning exercise, applying the above inclusion and exclusion criteria and a drop-out rate of 20%, we calculated that 19 out of 107 patients would be included.

1. **Course of studies (procedure)**

Procedure for informing and obtaining consent

The subjects will be informed about the study verbally as well as in writing using the enclosed participant information sheet. The subjects document their consent in writing using the enclosed consent form. The signed consent form (see enclosure) is a prerequisite for the study. There will be no remuneration for the participants.

Recording the target variables pre- and post (examinations, measurements, data collection)

- Gait analysis by means of sensory measurements and Gait Speed Test under different conditions
  - Walk Ratio
- Step length
- Step width
- Gear speed (normal/maximum/dual-task)
- Reserve capacity
- Timed "Up & Go" test
- Short Physical Performance Battery (SPPB)
- Balance test
- Walking test
- Chair Rise Test
- Quality of life questionnaire (EuroQuol)
- Questionnaire to assess fear of falling (FES-I)
- Test battery for attention diagnostics (TAP) and Digit-Span for recording simple verbal memory skills

During two measurement sessions (pre- and post) data will be collected using questionnaires of life satisfaction (EuroQuol) and fear of falling (FES-I). In addition, 8 gaits under 3 conditions (2x normal walking, 2x fast walking, 1x walking under dual-task condition with one test run each) will be recorded using a sensory measurement method (GAITRite or comparable) and the Gait Speed Test. Furthermore the Short Physical Performance Battery is carried out as a mobility test. In addition a computer-assisted measurement (TAP) of four attention parameters (simple reaction processing speed, divided attention capacity, cognitive switching capacity, working memory capacity) as well as a verbally derived task to record the simple memory span (digit span) will be carried out. Necessary socio-demographic data for the evaluation of the collected parameters are taken from the patient's file, as well as main and secondary diagnoses, medication and the geriatric assessment with, among others, the Timed "Up & Go" test and MMST.

Table 1 Data collection

| **No.** | **Parameter** | **Measuring time**  **Start (B),**  **End (E)** | **Standard in the hospital (S) /**  **Addition (Z)** | **Test** |
| --- | --- | --- | --- | --- |
| 1 | Characteristics of the participants (e.g. socio-demographic data, medication, diagnosis, geriatric assessment). | B | S | Patient file |
| 2 | Gait parameters | B, E | Z, S | GAITRite, Gait Speed |
| 3 | Physical parameters | B, E | Z, S | Short Physical Performance Battery  Timed "Up & Go" test |
| 4 | Quality of life | B, E | Z | EuroQuol |
| 5 | Fear of falling | B, E | Z | FES-I |
| 6 | Cognitive impairment | B | S | MMST |
| 7 | Attention and executive functions | B, E | Z | TAP |
| 8 | Immediate memory span and working memory | B, E | Z | TAP, NAI, WAIS-IV |

Randomisation

Randomisation is done by randomly assigning room numbers on the day of admission. Study participants who are in a room with an even room number are assigned to the control group. Study participants who are in a room with an odd room number will be assigned to the intervention group.

The department for geriatric early rehabilitation has 89 beds, of which 44 beds are in rooms with even numbers and 45 beds in rooms with odd numbers. Of the total of 6 single rooms, 5 are allocated to odd numbers, resulting in only a slight imbalance in the distribution of beds between even and odd room numbers.

By implementing simple randomisation, subjects are randomly assigned to a group without the possibility of manipulation of the assignment by a person involved in the study. Randomisation by room number has the additional advantage that the room neighbours cannot exchange information about their training programme. If a significant imbalance in group size occurs early in the study, the randomisation strategy will be adjusted. After a group has reached the target group size, study participants will be assigned to the incomplete group in the order of their inclusion in the further course.

Time schedule (appointments) and study duration for the subjects/patients (flow chart)

On the day of admission, a medical check is carried out for inclusion and exclusion criteria, the Mini Mental Status Test is carried out by trained staff and the consent form for participation and data protection is obtained.

All other cognitive tests and the questionnaires on fear of falling and life satisfaction will be carried out at the latest on the third day and, if possible, in one session with an approximate duration of 60 minutes at the beginning and again at the end of the study participation. If participants are unable to complete cognitive tests and questionnaires in one session for health or personal reasons, they can be split into two sessions. In this case, the cognitive testing is always carried out contiguously in order to create comparable conditions for the test persons.

The gait analysis using the GAITRite system as well as the Gait Speed Test and the Short Physical Performance Battery (SPPB) mobility test take about 30 minutes. This is done consecutively and at the latest on the third day after admission and as a re-test at the end of the study participation.

This results in the following chronological sequence

- Admission day: Checking for inclusion and exclusion criteria, performing the Mini Mental Status Test, informing and consenting
- Up to the third day of the stay: 60 minutes of cognitive testing including completion of questionnaires and 30 minutes of gait and mobility measurement.
- Physiotherapeutic treatment in the control group or participation in the SSE intervention as a combination of SSE and "Usual care" in the intervention group over the duration of the geriatric early rehabilitation "Usual care".
- Re-testing: 60 minutes of cognitive testing including completion of questionnaires and 30 minutes of gait and mobility measurement.

Intervention

Both groups get the same number and time in the training sessions overall for comparability.

The control group receives "routine physiotherapy" (usual care) 5 times a week for about 30 minutes per session.

The intervention group receives "routine physiotherapy" twice a week and SEE training three times a week for 30 minutes. The SSE training and the "routine physiotherapy" alternate. This is carried out over the entire period of geriatric early rehabilitation and so the number of training units may vary depending on the length of stay, but the ratio "routine physiotherapy”/ SSE+ remains the same. In SEE training, an increasing training load is used according to a structured training plan. At the beginning of the session, the subjects are familiarised with the carpet and learn how to walk across it. In the following, step patterns from the basic level are learned and increased in complexity/speed? according to the individual abilities. Step patterns are provided from each difficulty category, which can then be selected individually. The important thing here is to increase after successfully learning a pattern. The step patterns and classification into requirement levels were taken from the original Shigematsu study (2006 &2008) and a selection of patterns to be used was made within our research group.

After familiarisation with the training programme and confident learning of a pattern, a dual task is added. The subjects are asked to complete an additional cognitive task while running the learned pattern.

Examples of the requirement levels of the step patterns

Basic level:


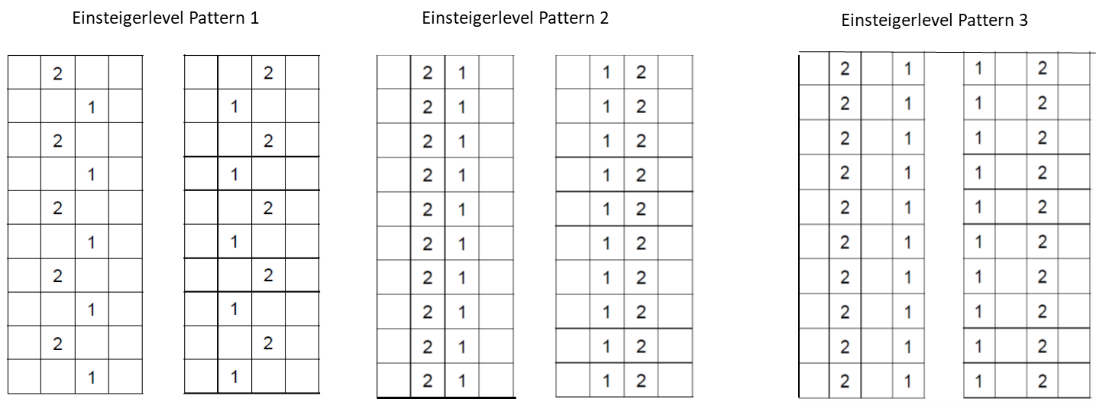

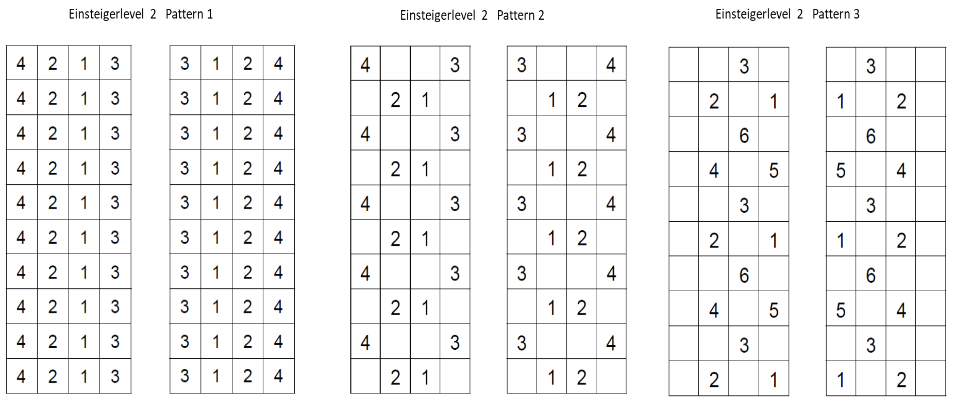


Total duration of the study

For the test persons, the study duration is approx. 3 weeks in total. The study is planned for a duration of 18 months.

1. **Risk-Benefit assessment (safety aspects)**

All study-related risks

Fundamental risks exist in the following areas of work:

- The functional tests (light muscle injuries e.g. bruises, strains)
- Speed measurements (GAITRite system or Gait Speed Test) (falls)

The methods used are standardised and established in practice. Participants are informed about the existing risks in the participant information. Gait speed measurements and functional tests are carried out by appropriately trained, expert personnel in scrupulous compliance with safety standards and test protocols, so that the basic risks are low. During recruitment strict adherence to exclusion criteria is observed and overtaxing of participants is unlikely to occur.

In the training programme both groups (intervention and control group) may experience slight muscle soreness. However, the trainers in both groups are trained physiotherapists who lead the rehabilitation programmes for the geriatric patients in the daily hospital routine and thus have many years of experience in the mobilisation of geriatric patients.

Benefits associated with the study

Up to now SSE has not been used in inpatient geriatric early rehabilitation. The study aims to investigate the effect of SSE training compared to "care as usual" physiotherapeutic treatment on gait parameters, executive functions, fear of falling and subjective life satisfaction.

The benefit associated with the study is scientific evidence of a physiotherapeutic training programme. The research question is whether better outcomes for patients in inpatient geriatric early rehabilitation can be achieved in the functional areas mentioned when physical and cognitive skills are trained simultaneously. Our study will go a long way in establishing whether the implementation of SSE in a typical hospital setting is effective and efficient as SSE can be implemented with little time and financial effort, would have a positive effect on duration of hospital stay and can also be continued by patients in their home environment, regardless of weather and with little risk of injury.

The scientific knowledge gained could be used to inform training in geriatric early rehabilitation and would contribute to the further development of this setting.

Discontinuation criteria (for the individual participant or for the entire study)

The examinations will be stopped at the patient's request, regardless of the reason. Fatigue or exhaustion of the patient visible to the test administrator will also lead to the termination of the examination.

1. **Data analysis**

After descriptive processing of the data, hypotheses are generated which are to be tested confirmatively in a second step using interferential statistical methods.

1. **Data management and data protection**

Data acquisition, storage

The data are processed pseudonomously. The data is stored at Klinikum Bremerhaven Reinkenheide gGmbH under standardised quality and security conditions. Only the direct staff of the project have access to the data.

The data is recorded in paper and digital form and kept for at least 10 years. After the end or termination of the study, the documents are stored in the archive of the clinic.

Data sharing

No data transfer is required. All data are evaluated and processed internally at the clinic.

See also attached data protection declaration with revocation and data deletion.

1. **Handling of biomaterials: Not applicable**
2. **Proband insurance (if applicable): No**

**Literature**

Al-Yahya, E., Dawes, H., Smith, L., Dennis, A., Howells, K., & Cockburn, J. (2011). Cognitive motor interference while walking: a systematic review and meta-analysis. *Neuroscience & Biobehavioral Reviews*, *35*(3), 715-728.

Ball, K., Berch, D. B., Helmers, K. F., Jobe, J. B., Leveck, M. D., Marsiske, M., & ACTIVE Study Group. (2002). Effects of cognitive training interventions with older adults: a randomized controlled trial. *Jama*, *288*(18), 2271-2281.

Bishnoi, A., Holtzer, R., & Hernandez, M. E. (2021). Brain Activation Changes While Walking in Adults with and without Neurological Disease: Systematic Review and Meta-Analysis of Functional Near-Infrared Spectroscopy Studies. *Brain sciences*, *11*(3), 291.

Brand, M., & Markowitsch, H. J. (2004). Frontalhirn und Gedächtnis im Alter. *NeuroGeriatrie*, *1*, 1-11.

Cabeza, R., Anderson, N. D., Locantore, J. K., & McIntosh, A. R. (2002). Aging gracefully: compensatory brain activity in high-performing older adults. *Neuroimage*, *17*(3), 1394-1402.

Cabeza, R., Daselaar, S. M., Dolcos, F., Prince, S. E., Budde, M., & Nyberg, L. (2004). Task-independent and task-specific age effects on brain activity during working memory, visual attention and episodic retrieval. *Cerebral cortex*, *14*(4), 364-375.

Colcombe, S., & Kramer, A. F. (2003). Fitness effects on the cognitive function of older adults: a meta-analytic study. *Psychological science*, *14*(2), 125-130.

Falck, R. S., Davis, J. C., Best, J. R., Crockett, R. A., & Liu-Ambrose, T. (2019). Impact of exercise training on physical and cognitive function among older adults: a systematic review and meta-analysis. *Neurobiology of aging*, *79*, 119-130.

Falck, R. S., Landry, G. J., Best, J. R., Davis, J. C., Chiu, B. K., & Liu-Ambrose, T. (2017). Cross-sectional relationships of physical activity and sedentary behavior with cognitive function in older adults with probable mild cognitive impairment. *Physical therapy*, *97*(10), 975-984.

Fisseha, B., Janakiraman, B., Yitayeh, A., & Ravichandran, H. (2017). Effect of square stepping exercise for older adults to prevent fall and injury related to fall: systematic review and meta-analysis of current evidences. *Journal of exercise rehabilitation*, *13*(1), 23.

Hausdorff, J. M., Yogev, G., Springer, S., Simon, E. S., & Giladi, N. (2005). Walking is more like catching than tapping: gait in the elderly as a complex cognitive task. *Experimental brain research*, *164*(4), 541-548.

Hedden, T., & Yoon, C. (2006). Individual differences in executive processing predict susceptibility to interference in verbal working memory. *Neuropsychology*, *20*(5), 511.

Holtzer, R., Ross, D., & Izzetoglu, M. (2020). Intraindividual variability in neural activity in the prefrontal cortex during active walking in older adults. *Psychology and Aging*, *35*(8), 1201.

Holtzer, R., Verghese, J., Xue, X., & Lipton, R. B. (2006). Cognitive processes related to gait velocity: results from the Einstein Aging Study. *Neuropsychology*, *20*(2), 215.

Jadczak, A. D., Makwana, N., Luscombe-Marsh, N., Visvanathan, R., & Schultz, T. J. (2018). Effectiveness of exercise interventions on physical function in community-dwelling frail older people: an umbrella review of systematic reviews. *JBI Evidence Synthesis*, *16*(3), 752-775.

Kearney, F. C., Harwood, R. H., Gladman, J. R., Lincoln, N., & Masud, T. (2013). The relationship between executive function and falls and gait abnormalities in older adults: a systematic review. *Dementia and geriatric cognitive disorders*, *36*(1-2), 20-35.

Logan, J. M., Sanders, A. L., Snyder, A. Z., Morris, J. C., & Buckner, R. L. (2002). Under-recruitment and nonselective recruitment: dissociable neural mechanisms associated with aging. *Neuron*, *33*(5), 827-840.

Lundin-Olsson, L., Nyberg, L., & Gustafson, Y. (1997). Stops walking when talking as a predictor of falls in elderly people. *Lancet*, *349*(9052), 617.

Montero‐Odasso, M., Verghese, J., Beauchet, O., & Hausdorff, J. M. (2012). Gait and cognition: a complementary approach to understanding brain function and the risk of falling. *Journal of the American Geriatrics Society*, *60*(11), 2127-2136.

Morrison, J. H., & Baxter, M. G. (2012). The ageing cortical synapse: hallmarks and implications for cognitive decline. *Nature Reviews Neuroscience*, *13*(4), 240-250.

Oswald, W. D., Gunzelmann, T., Rupprecht, R., & Hagen, B. (2006). Differential effects of single versus combined cognitive and physical training with older adults: the SimA study in a 5-year perspective. *European journal of ageing*, *3*(4), 179.

Reuter-Lorenz, P. A., Jonides, J., Smith, E. E., Hartley, A., Miller, A., Marshuetz, C., & Koeppe, R. A. (2000). Age differences in the frontal lateralization of verbal and spatial working memory revealed by PET. *Journal of cognitive neuroscience*, *12*(1), 174-187.

Sánchez-Arenas, R., Doubova, S. V., Bernabe-Garcia, M., Gregory, M. A., Mejía-Alonso, L. A., Orihuela-Rodríguez, O., & Shigematsu, R. (2020). Double-task exercise programmes to strengthen cognitive and vascular health in older adults at risk of cognitive decline: protocol for a randomised clinical trial. *BMJ open*, *10*(12), e039723.

Sebastião, E., McAuley, E., Shigematsu, R., Adamson, B. C., Bollaert, R. E., & Motl, R. W. (2018). Home-based, square-stepping exercise program among older adults with multiple sclerosis: results of a feasibility randomized controlled study. *Contemporary clinical trials*, *73*, 136-144.

Shellington, E. M., Reichert, S. M., Heath, M., Gill, D. P., Shigematsu, R., & Petrella, R. J. (2018). Results from a feasibility study of square-stepping exercise in older adults with type 2 diabetes and self-reported cognitive complaints to improve global cognitive functioning. *Canadian journal of diabetes*, *42*(6), 603-612.

Shigematsu, R., & Okura, T. (2006). A novel exercise for improving lower-extremity functional fitness in the elderly. *Aging clinical and experimental research*, *18*(3), 242-248.

Shigematsu, R., Okura, T., Nakagaichi, M., Tanaka, K., Sakai, T., Kitazumi, S., & Rantanen, T. (2008). Square-stepping exercise and fall risk factors in older adults: a single-blind, randomized controlled trial. *The Journals of Gerontology Series A: Biological Sciences and Medical Sciences*, *63*(1), 76-82.

Shigematsu, R., Okura, T., Sakai, T., & Rantanen, T. (2008b). Square-stepping exercise versus strength and balance training for fall risk factors. *Aging clinical and experimental research*, *20*(1), 19-24.

Shigematsu, R., Okura, T., Nakagaichi, M., & Nakata, Y. (2014). Effects of exercise program requiring attention, memory and imitation on cognitive function in elderly persons: a non-randomized pilot study. *J Gerontol Geriatric Res*, *3*(02), 147.

Smith, E., Cusack, T., & Blake, C. (2016). The effect of a dual task on gait speed in community dwelling older adults: A systematic review and meta-analysis. *Gait & posture*, *44*, 250-258.

Soshi, T., Andersson, M., Kawagoe, T., Nishiguchi, S., Yamada, M., Otsuka, Y., ... & Sekiyama, K. (2021). Prefrontal Plasticity after a 3-Month Exercise Intervention in Older Adults Relates to Enhanced Cognitive Performance. *Cerebral Cortex*.

Szameitat, A. J., Schubert, T., Müller, K., & Von Cramon, D. Y. (2002). Localization of executive functions in dual-task performance with fMRI. *Journal of cognitive neuroscience*, *14*(8), 1184-1199.

Swoboda, W., Sieber, C. Rehabilitation in der Geriatrie. *Internist* 51, 1254-1261 (2010).

Teixeira, C. V. L., Gobbi, S., Pereira, J. R., Vital, T. M., Hernandéz, S. S. S., Shigematsu, R., & Gobbi, L. T. B. (2013). Effects of square‐stepping exercise on cognitive functions of older people. *Psychogeriatrics*, *13*(3), 148-156.

Webber, S. C., Porter, M. M., & Menec, V. H. (2010). Mobility in older adults: a comprehensive framework. *The gerontologist*, *50*(4), 443-450.

Willis, S. L., Tennstedt, S. L., Marsiske, M., Ball, K., Elias, J., Koepke, K. M., & ACTIVE Study Group, F. T. (2006). Long-term effects of cognitive training on everyday functional outcomes in older adults. *Jama*, *296*(23), 2805-2814.

Woollacott, M., & Shumway-Cook, A. (2002). Attention and the control of posture and gait: a review of an emerging area of research. *Gait & posture*, *16*(1), 1-14.

World Health Organization. (2015). *World report on ageing and health*. World Health Organization.

Yeom, H. A., Fleury, J., & Keller, C. (2008). Risk factors for mobility limitation in community-dwelling older adults: a social ecological perspective. *Geriatric nursing*, *29*(2), 133-140.
