# Supplementary material for: Effects of Square-Stepping Exercise on cognitive function in early geriatric rehabilitation: A randomized controlled explorative study
Source: PLoS One. 2026 Jan 5;21(1):e0338695. doi: 10.1371/journal.pone.0338695 (PMC12768348; doi:10.1371/journal.pone.0338695)
Supplement: S2 Appendix — (DOCX) [file pone.0338695.s002.docx]

Akademisches Lehrkrankenhaus

der Universität Göttingen

Medizinische Klinik III
Geriatrie

**Dr. med. Helmut Ackermann**

Chefarzt

Innere Medizin- Geriatrie-

Nephrologie

Telefon: (0471) 299-3690

Telefax: (0471) 299-3743

Internet: www.klinikum-bremerhaven.de

Klinikum Bremerhaven, Postbrookstr. 103, 27574 Bremerhaven

**Step by Step: Eine randomisierte kontrollierte Pilotstudie zum Effekt von Square-Stepping Exercise in der stationären geriatrischen Frührehabilitation**

**Studienprotokoll Forschungsvorhaben**

1. **Studientitel**

Step by Step: Eine randomisierte kontrollierte Pilotstudie zu Square-Stepping Exercise in der stationären geriatrischen Frührehabilitation

1. **Zusammenfassung der Studie**

Die Mobilität im Alter wird maßgeblich durch die Gehfähigkeit und durch das kognitive Leistungsvermögen beeinflusst. Beide Aspekte stellen wichtige Determinanten für die physische Gesundheit und das psychische Wohlbefinden dar. Es ist erwiesen, das Gehen ein Zusammenspiel höherer kognitiver Funktionen erfordert, wobei die kognitive Anforderung beim Gehen im Alter steigt. Gleichzeitig sinkt jedoch die kognitive Leistungsfähigkeit im Alter. Dieses Zusammenspiel von physischer Aktivität und kognitivem Leistungsvermögen im höheren Alter wurde mehrfach im Rahmen des Dual-Task Paradigmas untersucht und konnte deutliche Interferenzen aufzeigen.

Eine Abnahme der Gehfähigkeit und der Gangsicherheit ist mit multiplen Einschränkungen und einer Abnahme der Selbstständigkeit vergesellschaftet. Auch wurde in Langzeitstudien ein erhöhtes Demenzrisiko bei klinischen Gangveränderungen abgeleitet.

Es gibt bereits eine Vielzahl an klinischen Untersuchungen zur Verbesserung der Mobilität durch physische Aktivität. Unter den Dachbegriff der physischen Aktivität fallen neben Sportarten auch gezieltes Training zur Verbesserung einzelner Funktionen. Studien zur physischen Aktivität beziehen sich häufig auf die Zunahme der funktionalen Fitness der unteren Extremität zur Sturzprophylaxe. So entwickelten Shigematsu und Okura das „Square-Stepping Exercise“ (SSE), das erstmals 2006 als gezieltes Training mit selbstständig lebenden Senioren erprobt wurde. Das Training wird auf einer Matte praktiziert, die in 40 Quadrate eingeteilt ist und basiert auf der Ausführung verschiedener Schrittmuster mit Ausfallschritten nach vorne, hinten und zur Seite mit aufsteigendem Schwierigkeitsgrad. Die Autoren verfolgten dabei das Ziel, durch ein Training sowohl das Sturzrisiko zu vermindern, als auch die funktionale Fitness der unteren Extremität zu verbessern. Nach dem Prinzip der proaktiven und reaktiven Reaktionsverbesserung soll durch das Training die Durchführung eines Korrekturschrittes zur Wiederherstellung des Gleichgewichts

infolge eines Stolperns optimiert werden, um Stürze zu verhindern. Durch Aktivierung von Agonisten und Antagonisten der unteren Extremität soll außerdem die Fitness verbessert werden.

Es gibt mittlerweile zahlreiche Studien zum Einfluss von körperlicher Aktivität auf die kognitiven Fähigkeiten bei älteren Menschen. Diese haben signifikante Effekte auf Exekutiv- und Gedächtnisfunktionen gezeigt. Vor allem wurden dabei Trainingsformen, wie Walking, Aerobic und Ausdauertraining untersucht. Diese Studien zeigen aber auch, dass gezielte körperliche Aktivität nur dann einen signifikanten Effekt auf die kognitive Leistungsfähigkeit hat, wenn im Training sowohl Anforderungen an körperliche als auch an die kognitive Leistungsfähigkeit gestellt werden.

Die Dual Task Anforderung ist im SSE-Training besonders hoch, weshalb diese Trainingsform sowohl körperliche als auch kognitive Aktivität vereint. Die Probanden müssen sich zunächst die Instruktion des Trainers merken, um die Muster korrekt ausführen zu können und anschließend die Instruktion motorisch umsetzen. Da sowohl Anforderungen an die motorische Umsetzung als auch an die Aufmerksamkeitsteilung und Arbeitsgedächtnisleistung durch das Training gestellt werden, wird postuliert, dass sich durch SSE-Training ein positiver Effekt auf die kognitive Leistungsfähigkeit erzielen lässt.

Bislang wurde das SSE wissenschaftlich ausschließlich an selbstständig lebenden Senioren untersucht. Dieser Aspekt veranlasst uns, dieses Übungsprogramm im Rahmen der stationären Frührehabilitation im Vergleich zur herkömmlichen Physiotherapie zu untersuchen. Die herkömmliche Physiotherapie beinhaltet neben Übungen zur Kräftigung der unteren Extremität häufig ein Gehtraining auf der Stationsebene und Übungen zum Erhalt alltagsrelevanter Aktivitäten, wie zum Beispiel das Treppe steigen.

Bereits seit einigen Jahren wenden wir in unserer geriatrischen Akutrehabilitation Elemente aus dem SSE an, jedoch ohne Messung der objektiven Verbesserung auf die die körperliche Funktion der unteren Extremität, die kognitiven Fähigkeiten oder die Mobilität. Zu dem Setting der stationären geriatrischen Frührehabilitation gibt es bislang keine wissenschaftlichen Untersuchungen, weshalb diese Studie als Pilotprojekt das SSE Training im Vergleich zur herkömmlichen Physiotherapie auf eine Verbesserung der körperlichen Funktion der unteren Extremität als primäres Outcome sowie auf die Exekutivfunktionen und die Mobilität untersuchen soll.

1. **Verantwortlichkeiten**

Studienleiterin

Dr. Svenja Tietgen

Abteilung für Geriatrie

Chefarzt Herr Dr. med. Helmut Ackermann

Klinikum Bremerhaven Reinkenheide gGmbH

Postbrookstraße 103

27574 Bremerhaven

Weitere Mitarbeiterin

Katja Fränzel

Abteilung für Geriatrie

Chefarzt Herr Dr. med. Helmut Ackermann

Klinikum Bremerhaven Reinkenheide gGmbH

Postbrookstraße 103

27574 Bremerhaven

Beteiligte Einrichtungen/Kooperationspartner

PD Dr. Ellen Freiberger

Institut für Biomedizin des Alterns FAU Erlangen-Nürnberg

Kobergerstr. 60

90408 Nürnberg

Professor Ryosuke SHIGEMATSU, Ph.D.

School of Health and Sport Science,

Chukyo University

101 Tokodachi, Toyota, Aichi,

Japan 470-0393

Prof. Dr. Tania Zieschang

Abteilung für Geriatrie

Carl von Ossietzky Universität Oldenburg

Fakultät VI Medizin und Gesundheitswissenschaften

Department für Versorgungsforschung

Ammerländer Heerstraße 140, Gebäude V04, Raum 118

26129 Oldenburg

Dr. Jessica Koschate

Abteilung für Geriatrie

Carl von Ossietzky Universität Oldenburg

Fakultät VI Medizin und Gesundheitswissenschaften

Department für Versorgungsforschung

Ammerländer Heerstraße 140, Gebäude V04, Raum 118

26129 Oldenburg

Geldgeber

Keine. Aktuell sind keine Drittmittel beantragt

1. **Wissenschaftlicher Hintergrund**

Mobilität im Alter ist eng assoziiert mit dem Erhalt der physischen und psychischen Gesundheit (Yeom, Fleury & Keller, 2008; Webber, Porter & Menec, 2010). Webber & Kollegen (2010) gingen dem Versuch einer Operationalisierung des Konzeptes von Mobilität im Alter nach und fassten fünf grundlegende Kategorien bestimmender Faktoren zusammen: Kognitive, psychosoziale, physische, ökologische und finanzielle Determinanten. Ein entscheidender Aspekt der physischen Determinante stellt die Gehfähigkeit dar. Zur weitestgehend unabhängigen Durchführung der Aktivitäten des täglichen Lebens ist außerdem die kognitive Leistungsfähigkeit entscheidend. Beide Variablen dienen maßgeblich dem Erhalt der Selbstständigkeit, wodurch die gesellschaftliche Teilhabe gefördert und die Lebensqualität älterer Menschen gesteigert wird.

Ähnlich wird in dem Bericht der Weltgesundheitsorganisation (WHO) zu Altern und Gesundheit aus 2015 der Fokus auf die Funktionalität als entscheidender Faktor für das gesunde Altern gelegt. Das Konzept „Healthy Ageing“ wird als Prozess der Entwicklung und Aufrechterhaltung der Funktionsfähigkeit mit dem Ziel des individuellen Wohlbefindens definiert. Als entscheidender Faktor dieser Funktionsfähigkeit wird die intrinsische Leistungsfähigkeit einer Person betrachtet, die sich aus den körperlichen und ebenso aus den geistigen Fähigkeiten zusammensetzt. Es wird angenommen, dass die Einschätzung der Funktionsfähigkeit ein besserer Prädiktor für ein positives Outcome im Alter ist, als einzelne Erkrankungen oder das Ausmaß der Multimorbidität.

Daraus lässt sich der hohe Stellenwert der Funktionalität, die sowohl auf körperlicher als auch auf geistiger Leistungsfähigkeit und deren Wechselwirkung beruht, ableiten.

Insbesondere zur Früherkennung von Demenz- und Sturzrisiken hat die Erforschung von Gangveränderungen im Alter in den vergangenen Jahren zunehmend Interesse gefunden. Gang- und Kognitionsparameter werden dabei nicht länger als zwei unabhängige Prozesse verstanden, vielmehr wird die Interaktion beider als wechselseitige Beziehung zueinander untersucht (Holtzer, Verghese, Xue & Lipton, 2006 Al-Yahya et al. 2011; Montero-Odasso et al. 2012, Cohen, Verghese & Zwerling, 2016). Die früher verbreitete Hypothese, beim Gehen handele es sich um eine stark überlernte, automatisiert rhythmische Bewegung wurde spätestens durch die Untersuchungen von Hausdorff und Kollegen (2005) widerlegt. In der Studie konnte gezeigt werden, dass das Gehen einem Zusammenspiel höherer kognitiver Funktionen, wie Wahrnehmung, Handlungsplanung, Aufmerksamkeits- und Gedächtnisprozesse bedarf. Die Autoren leiteten ab, dass auch das routinemäßige Gehen einer komplexen kognitiven Aufgabe entspricht, wodurch sich ein neuer Ansatz für die Behandlung von Gangverschlechterung und Sturzrisiko bei älteren Menschen ableiten lässt. Mit zunehmendem Alter ändern sich die Aufmerksamkeitsanforderungen an die Gang- und Gleichgewichtskontrolle, wodurch die kognitive Anforderung an das Gehen im Alter steigt (Woollacott & Shumway-Cook, 2002¸Hausdorff et al. 2005).

Im Zuge eines normalen Alterungsprozesses ergeben sich ebenso strukturelle und funktionelle Änderungen auf Hirnebene. Eine Modifikation dieser ergibt sich durch physiologische Abbauprozesse, aber auch durch Umstrukturierungsprozesse des alternden Gehirns. Mittlerweile wird von einer Wechselwirkung zwischen den strukturellen Änderungen durch natürliche Alterungsprozesse und sich ändernde kognitive Anforderungen an das Gehirn im Alter ausgegangen (Brand & Markowitsch 2004). Alternsbedingte Abbauprozesse zeigen sich in einigen Strukturen zu einem früheren Zeitpunkt stärker, in anderen weniger stark bis gar nicht ausgeprägt. Insbesondere in den präfrontalen und medial temporalen Strukturen zeigen sich die deutlichsten Alterseffekte. Als neuropsychologisches Korrelat äußert sich dies in einer Minderung exekutiver, aber auch mnestischer Funktionen (Hedden & Yoon, 2006; Morrison & Baxter, 2012).

Elektrophysiologische Untersuchungen konnten eine veränderte neuronale Aktivität bei älteren Probanden im Vergleich zu jüngeren Probanden nachweisen, die eine neuronale Umstrukturierung im Alter nahelegen. Es konnte gezeigt werden, dass ältere Probanden andere neuronale Schleifen zur Bewältigung von Testanforderungen nutzen. Es konnte insbesondere eine stärkere präfrontale Aktivierung nachgewiesen werden (Cabeza et al. 2002; Cabeza et al. 2004). Die aktuelle Forschung zu veränderter neuronaler Aktivität im Alter fokussiert auf die kognitive Reservekapazität, die unter anderem mit den Strukturen des präfrontalen Kortex in Verbindung gebracht wird. So deutet eine aktuelle Längsschnittstudie von Soshi und Kollegen (2021) darauf hin, dass schon kurzfristige kognitive Trainingsinterventionen über drei Monate eine präfrontale Plastizität im Zusammenhang mit der kognitiven Leistung bei älteren Erwachsenen induzieren können.

In der jüngeren Forschung wurde vermehrt der Frage nachgegangen, welche neuronalen Mechanismen der Gangkontrolle bei älteren Menschen zugrunde liegen. So untersuchten beispielsweise Harada, Miyai, Suzuki und Kobota (2009) mittels funktioneller Nahinfrarotspektroskopie (fNIRS) welche kortikalen Aktivierungsmuster mit der Ganggeschwindigkeit in Abhängigkeit mit der Gehfähigkeit bei älteren Menschen assoziiert sind. Die Ergebnisse legen nahe, dass die Steuerung der Ganggeschwindigkeit durch den linken präfrontalen Kortex, die supplementär-motorische Rinde sowie den sensomotorischen Kortex gesteuert wird. Mit höherem Alter geht eine erhöhte präfrontale Aktivierung bei kognitiven Anforderungen, unter anderem an die räumliche und verbale Arbeitsgedächtnisleistung, einher (Reuter-Lorenz et al, 2000). Die Meta-Analyse zu fNIRS-Studien zur Änderung der Gehirnaktivität beim Gehen bei Erwachsenen mit und ohne neurologische Erkrankungen von Bishnoi, Holtzer und Hernandez (2021) konnte die Annahme einer signifikanten Zunahme der Gehirnaktivierung im präfrontalen Kortex beim Gehen unter Dual-Task Bedingungen im Vergleich zum Stehen und einfachen Gehen bestätigen. Gleichzeitig wird angenommen, dass eine stärkere linksdominante präfrontale Aktivierung durch eine mit dem Altern bedingten Verlust des kognitiven Leistungsvermögens assoziiert ist (Logan et al., 2002). Der durch das Altern bedingte Rückgang der Gangkapazität bei gleichzeitigem Rückgang exekutiver Funktionen mit Beteiligung des präfrontalen Kortex verdeutlicht die Wechselwirkung zwischen der Gehfähigkeit und der exekutiven Leistungsfähigkeit. Der systematischen Übersicht von Kearney und Kollegen (2013) kann entnommen werden, dass sich durch das Altern bedingte Veränderungen der Exekutivfunktionen und Informationsverarbeitungsgeschwindigkeit auf die Gehfähigkeit und das Sturzrisiko älterer Menschen auswirken (Kearney et al. 2013). Basierend auf der Annahme, dass die kognitive Leistungskapazität bei älteren Menschen begrenzt ist, werden mit dem Dual-Task-Paradigma in klinischen Studien Interferenzen zwischen Gehen und kognitiven Funktionen detektiert. So zeigten Holtzer, Ross und Izzetoglu (2020) kürzlich mittels fNIRS beim aktiven Gehen bei älteren Erwachsenen eine positive Korrelation der Variabilität in der neuronalen Aktivität im präfrontalen Kortex und der Gangleistung, die sich ausschließlich unter Dual-Task Bedingungen abbilden ließ.

Die Testleistungen in Dual-Task-Aufgaben dienen der Quantifizierung der Exekutivfunktionen, die als maßgeblich für die kognitive Reservekapazität angesehen werden (Herman et al. 2010) und nach aktueller Studienlage mit bildgebenden Verfahren mit einer erhöhten Aktivität im dorsolateralen präfrontalen Kortex einhergehen (Herman et al, 2010; Szameitat, 2002). Studien mit Dual-Task-Anforderungen zeigen dabei eine Interferenz von Ganggeschwindigkeit und exekutiven Funktionen (Smith, Cusack & Blake, 2016). In ihrer Metaanalyse zu kognitiv motorischer Interferenz während des Gehens zeigten Al-Yahya et al. (2011) einen maßgeblichen Effekt verschiedener kognitiver Aufgaben auf die Ganggeschwindigkeit. Die Meta-Regressionsanalyse wies dabei auf einen starken Zusammenhang zwischen Alter und Verminderung der Gehgeschwindigkeit sowie zwischen dem kognitiven Leistungsvermögen und der Gehgeschwindigkeit unter Dual-Task-Bedingungen hin. Das Ausmaß der Interferenz kann bis hin zu einer Unterbrechung der motorischen Aufgabe bei erhöhter kognitiver Anforderung kommen. Als „stops walking when talking“-Phänomen ist diese maximale Interferenz als Prädiktor für Stürze im stationär geriatrischen Setting bekannt geworden (Lundin-Olsson, Nyberg & Gustafson 1997).

Der Effekt von einem körperlichen Training auf die kognitive Leistungsfähigkeit und körperlichen Funktionsparameter, wie Kraft, Mobilität, Gang und Gleichgewicht, bei älteren Menschen wurde bereits in mehreren systematischen Übersichtsarbeiten diskutiert. Diese weisen übereinstimmend darauf hin, dass ein Mehrkomponententraining, einschließlich Kraft-, Aerobic-, Gleichgewichts- und Flexibilitätstraining zur Verbesserung der körperlichen Funktionsparameter am besten geeignet zu sein scheinen. Kein Konsens besteht bislang darüber, welche Trainingsmerkmale, in Bezug auf Art, Dauer, Intensität, Häufigkeit und Kombination, langfristig am effektivsten sind. In einer Querschnittsstudie untersuchten Falck et al. (2017) den Zusammenhang von körperlicher Aktivität und der Entstehung von Mild Cognitive Impairment (MCI) und kamen zu der Annahme, dass >150Minuten/ Woche körperliche Aktivität mit besseren kognitiven Fähigkeiten einhergehen. Andersherum muss untersucht werden, ob MCI zu neurobiologischen Veränderungen führt, die eine Reduktion der körperlichen Aktivität nach sich zieht. Oswald und Kollegen (2006) untersuchten in einer Längsschnittstudie über 5 Jahre den Langzeiteffekt von kognitivem und körperlichem Training bei selbstständig lebenden Senioren im Alter von 75 bis 93 Jahren. Signifikante Trainingseffekte nach 5 Jahren im Vergleich zur Baseline konnten für die Probandengruppe gemessen werden, die ein kombiniertes kognitives und körperliches Training erhielten. Das kürzlich erschienene Cochrane Review von Jadczak et al. (2018) inkludierte sieben systematische Übersichtsarbeiten zur Untersuchung der Effektivität von körperlichem Training auf die physische Funktionalität von gebrechlichen älteren Menschen. Folglich können Mehrkomponententrainings, einschließlich Widerstands-, Aerobic-, Gleichgewichts- und Flexibilitätsaufgaben, für gebrechliche ältere Erwachsene zur Steigerung der Muskelkraft, Ganggeschwindigkeit, Gleichgewicht und körperlichen Leistungsfähigkeit empfohlen werden.

Inzwischen gilt als gesichert, dass eine regelmäßige kognitive Stimulation einen positiven Effekt auf die kognitive Leistungsfähigkeit im höheren Lebensalter hat (Ball et al. 2002; Willis et al. 2006). Jedoch zeigt sich ein heterogenes Bild bei der Quantifizierung kognitiver Fähigkeiten. Colombe & Kramer (2003) untersuchten beispielsweise in einer Metaanalyse den Effekt von Aerobic Training auf die kognitive Leistungsfähigkeit bei Senioren. Insgesamt wurden 18 Studien eingeschlossen, die zwischen 1966 und 2001 publiziert wurden. Unabhängig von der Art der kognitiven Aufgabe, die den jeweiligen Studien zugrunde lag und unabhängig von den Personenmerkmalen der Probanden wurde die kognitive Leistungsfähigkeit durch das angewandte Fitnesstraining um durchschnittlich 0,5 Standardabweichungen gesteigert. Die Autoren zeigten, dass die Trainingseffekte robust, jedoch von selektivem Nutzen für kognitive Funktionsbereiche sind. Die Analyse ergab den größten prozessspezifischen Nutzen von körperlichem Fitnesstraining auf exekutive und kognitive Kontrollprozesse. Colombe und Kramer kamen zu dem Schluss, dass diejenigen kognitiven Funktionsbereiche, für die in früheren Studien ein altersassoziierter Leistungsabfall belegt wurde, durch Aerobic Training verbessert werden können.

Die Metaanalyse zur Auswirkung von physischer Aktivität auf die körperlichen und kognitiven Funktionen bei älteren Erwachsenen von Falck et al. (2019) bestätigen den, in den vorangegangenen Studien, postulierten signifikanten Effekt von körperlichem Training sowohl für die körperlichen, als auch für die kognitiven Funktionen.

Das Square-Stepping Exercise beinhaltet eine hohe Dual Task Anforderung, weshalb eine Verbesserung des kognitiven Leistungsvermögens durch das Training postuliert wird (Teixeira et al. 2013). Das ursprünglich von Shigematsu und Okura in Japan entwickelte Programm hatte primär zum Ziel, die funktionelle Fitness der unteren Extremität zu verbessern, um die Gehfähigkeit älterer Menschen in der Allgemeinbevölkerung zu verbessern und durch eine höhere funktionelle Fitness das Sturzrisiko zu minimieren (Shigematsu & Okura, 2006; Shigematsu et al. 2008b).

SSE wird auf einer dünnen Matte, die in 40 Quadrate unterteilt ist, praktiziert. Dabei werden Schrittmuster, je nach Schwierigkeitsgrad mit Ausfallschritten nach vorne, hinten und zur Seite gelaufen. Die Muster können auch im Zehen- oder Fersengang gelaufen werden. Innerhalb des Trainings steigt die Komplexität der Laufmuster. Die Laufmuster dienen nach dem Prinzip der proaktiven und reaktiven Reaktionsverbesserung dazu, die Wiederherstellung des Gleichgewichts nach einem Stolpern durch einen Korrekturschritt zu verbessern.

**Stand der Forschung mit Ableitung der Fragestellung (Rationale)**

Fisseha und Kollegen 2017 lieferten in ihrer systematischen Übersicht und Meta-Analyse zur Wirksamkeit von SSE hinsichtlich Sturzprophylaxe und Sturzverletzungen einen Überblick zur Studienlandschaft. SSE erwies sich als wirksam zur Verminderung von Sturzangst sowie zur Verbesserung des subjektiv wahrgenommenen Gesundheitszustandes älterer Personen. Die tatsächliche Überlegenheit gegenüber anderen Interventionen wird jedoch auch aufgrund der noch sehr dünnen Studienlage kontrovers betrachtet.

Die Literaturrecherche zum Einfluss von SSE auf die kognitive Leistungsfähigkeit ergab gegenwärtig zwei klinische Untersuchung mit dem Fokus auf die Kognition. Teixeira und Kollegen (2013) untersuchten erstmals die Auswirkung eines 16-wöchigen SSE Trainings auf die kognitive Leistungsfähigkeit älterer Menschen. Die Autoren schlussfolgerten, dass SSE, als eine Form der globalen kognitiven Stimulation, einen positiven Einfluss auf das kognitive Leistungsvermögen, insbesondere in der fokussierten Aufmerksamkeitsleistung sowie in der kognitiven Flexibilität, älterer Menschen hat. Shigematsu und Kollegen (2014) untersuchten den Effekt eines täglichen SSE-Trainings bei gesunden Senioren im häuslichen Umfeld im Vergleich zu einem täglichen SSE-Training alle 14 Tage über einen Zeitraum von sechs Monaten auf die Performanz in 5 kognitiven Tests zu Exekutiv- und Gedächtnisfunktionen. In einem Testverfahren zu Exekutivfunktionen konnte eine signifikante Verbesserung der Testleistung bei durchgängigem SSE-Training erzielt werden. Die Gedächtnisparameter wurden in beiden Gruppen verbessert. Die Quantifizierung der kognitiven Parameter in der Pilotstudie hat jedoch eher einen globalen Charakter, was den geringen Effekt in den übrigen drei Testverfahren erklären könnte.

Die bisherige Studienlage legt nahe, dass durch die regelmäßige Anwendung eines SSE Training sowohl eine kognitive Stimulation als auch ein körperliches Training erfolgen, wodurch gleichzeitig eine Verbesserung der Gehfähigkeit sowie der kognitiven Leistungsfähigkeit erzielt werden kann. Dabei ist SSE in seiner Durchführung simpel und ökonomisch, da lediglich eine flache Trainingsmatte benötigt wird. Es ist körperlich weniger anstrengend, als Aerobic, Kraft- oder Fitnesstraining und kann im häuslichen Umfeld praktiziert werden. Somit bietet SSE gleichsam eine Alternative zum Outdoor Training bei schlechten Witterungsverhältnissen, eine Alternative zu externen Sportangeboten für weniger mobile Senioren und eine Alternative zu körperlich anstrengender Aktivität für Senioren in reduzierterem Allgemeinzustand.

Inzwischen wurden auch vereinzelt Machbarkeitsstudien zur Anwendung von SSE zur Verbesserung klinischer Symptome bei verschiedenen Krankheitsbildern, wie multipler Sklerose (Sebastião et al. 2018) und Diabetes (Shellington et al. 2018) durchgeführt. Die Ergebnisse unterstützten die Machbarkeit und Akzeptanz für das Training. Auch zeigten sich Hinweise auf eine mögliche Verbesserung exekutiver Funktionen bei Erwachsenen mit Diabetes Typ 2.

Derzeit läuft in Mexiko eine doppelblinde RCT, die den Effekt von SSE Training bei 60-65- Jährigen auf vaskuläre und kognitive Funktionen untersucht (Sanchez-Arenas 2020), deren Ergebnisse noch ausstehend sind.

Nach aktueller Studienlage wurden im Rahmen klinischer Studien zur Reduktion des Sturzrisikos oder zur Steigerung kognitiver und physischer Funktion älterer Menschen, das Training bislang häufig für selbstständig lebende Senioren im häuslichen Umfeld konzipiert, bei denen die Studienteilnehmer über Medien rekrutiert wurden. Für das stationäre geriatrische Setting hingegen wurden bislang kaum/keine Trainingskonzepte zur Verbesserung der Gehfähigkeit und kognitiven Leistungsfähigkeit konzipiert. Auch ist wenig Forschung bezüglich der Wechselwirkung von kognitivem Training und körperlichem Training bei geriatrischen Patienten betrieben worden.

Der stationären geriatrischen Rehabilitation liegt als übergeordnetes Rehabilitationsziel die Verbesserung der Mobilität zugrunde, das die Verbesserung der sozialen Interaktion sowie Vermeidung, bzw. Verminderung der Abhängigkeit von Pflegepersonen zum Erhalt der Selbstständigkeit einschließt (Swoboda & Sieber, 2010). Im Rahmen der multidisziplinären Behandlung im geriatrischen Team erfolgt die körperliche Mobilisierung der Patienten hauptsächlich durch die physiotherapeutische Behandlung. Neben Anwendungsverfahren, wie manuellen Therapien, physiotherapeutische Schmerztherapie und Lymphdrainage kommt funktionelles Training zur Steigerung von Kraft, Ausdauer, Gleichgewicht, Stand- und Gangsicherheit zum Einsatz, das bislang jedoch keiner Standardisierung unterliegt.

Gemäß dem Konzept „Healthy Ageing“ der WHO (2015) sollte ein Training zur Förderung und Aufrechterhaltung der Mobilität älterer Menschen gleichsam auf die physische, wie auch auf die kognitive Funktion abzielen.

Aufgrund der hohen Dual-Task-Anforderung, die das SSE beinhaltet, und der als erwiesen anzunehmenden Wirksamkeit des Trainings bezüglich der Verbesserung der funktionellen Fitness der unteren Extremität (Shigematsu & Okura, 2006; Shigematsu et al. 2008; Shigematsu et al. 2008b), wird ein Effekt auf die Gehfähigkeit und die kognitive Leistungsfähigkeit im Funktionsbereich der Exekutivfunktionen und des Arbeitsgedächtnisses angenommen.

Durch die Anwendung eines einheitlichen SSE-Trainingsplans am Patientenkollektiv der stationären geriatrischen Frührehabilitation soll die Pilotstudie „Step by Step“ einen wesentlichen Erkenntnisgewinn liefern. Im Gegensatz zu den bisherigen Studien zum Effekt von SSE an selbstständig lebenden Senioren, bietet dieses Setting ein breit gefächertes Patientenkollektiv, welches für eine repräsentative Stichprobe steht. Bislang wurden Senioren über Medien rekrutiert. Dieser Rekrutierungsweg setzt sowohl voraus, dass die Probanden über ein höheres Maß an kognitiver und physischer Fitness und eine höhere Mobilität verfügen sowie eine intrinsische Motivation an der Steigerung der körperlichen Leistungsfähigkeit bereits vorbestehend ist. Senioren, denen Medien weniger zugänglich sind oder, die über ein geringeres Maß an Mobilität verfügen, werden über diesen Rekrutierungsweg weniger erreicht.

Bislang wurde der Effekt eines SSE Trainings auf die kognitive Leistungsfähigkeit zumeist auf Basis eines globalen Ansatzes untersucht. So kamen häufig Screeninginstrumente, wie der Mini Mental Status Test, das Montreal Cognitive Assessment oder Untertests aus verschiedenen Testbatterien zum Einsatz. Eine dezidierte Messung einzelner kognitiver Funktionsbereiche ist über diese Instrumente jedoch nicht möglich. Auch sind Screeninginstrumente in der Regel nicht alters-, bildungs- oder geschlechtsadjustiert und anfälliger für tagesaktuelle Schwankungen und andere Einflussfaktoren. Statistisch signifikante Veränderungen lassen sich dadurch nicht valide messen. Hierbei stellt auch der Umstand, dass es sich beim Begriff der Exekutivfunktionen um ein heterogenes Konstrukt handelt, eine besondere Herausforderung dar. Die Ergebnisse aus klinischen Studien zum wechselseitigen Effekt von körperlicher Aktivität und Exekutivfunktionen sind durch die Betrachtung unterschiedlicher Domänen, die die Autoren den Exekutivfunktionen zuordnen, wenig vergleichbar. In dieser Pilotstudie wird daher eine dezidierte und standardisierte Messung verschiedener Subdomänen der Exekutivfunktionen erfolgen, um den Effekt der Dual-Task-Anforderung aus dem SSE-Training gezielt einzelnen Subdomänen zuordnen zu können und mögliche Zusammenhänge dieser aufzudecken. Mit dem Ziel der Steigerung des Rehabilitationserfolges in der stationären geriatrischen Frührehabilitation, können diese Erkenntnisse schließlich in die therapeutische Praxis implementiert werden.

In der Pilotstudie „Step by Step“ soll daher ein SSE Training im Rahmen der stationären geriatrischen Frührehabilitation im Vergleich zur herkömmlichen physiotherapeutischen Behandlung mit dem primären Outcome der Verbesserung der körperlichen Funktion der unteren Extremität untersucht werden. Darüber hinaus könnte die Durchführung der Studie Aufschluss darüber geben, ob sich eine signifikante Verbesserung auf die Exekutivfunktionen und die Mobilität nach einem zwei- bis dreiwöchigen SSE Training messen lässt. Weiter werden Veränderungen der Sturzangst und der subjektiven gesundheitsbezogenen Lebensqualität erhoben. Die Quantifizierung der kognitiven Parameter erfolgt dezidiert und standardisiert zu einzelnen Funktionsbereichen der Exekutivfunktionen, um spezifische Effekte des SSE-Trainings besser darstellen und nachvollziehen zu können. Die Durchführung dieser Pilotstudie könnte somit Aufschluss darüber geben, ob sich durch die Implementierung eines SSE Trainings in die stationäre geriatrische physiotherapeutische Behandlung ein größerer Rehabilitationserfolg hinsichtlich der Mobilität für die Patient:innen erzielen lässt. Dies könnte zu einer Optimierung des Behandlungskonzeptes beitragen und würde neue Ansatzpunkte für die Forschung eröffnen.

1. **Studienziele**

Forschungsfrage 1: Lässt sich durch eine Kombinationsbehandlung aus SSE und herkömmlicher Physiotherapie im Vergleich zur Kontrollgruppe mit ausschließlich herkömmlicher Physiotherapie eine Veränderung der körperlichen Funktion der unteren Extremität messen?

Forschungsfrage 2: Lässt sich durch eine Kombinationsbehandlung aus SSE und herkömmlicher Physiotherapie im Vergleich zur Kontrollgruppe mit ausschließlich herkömmlicher Physiotherapie eine Leistungssteigerung in den Exekutivfunktionen erzielen?

Forschungsfrage 3: Lässt sich durch eine Kombinationsbehandlung aus SSE und herkömmlicher Physiotherapie im Vergleich zur Kontrollgruppe mit ausschließlich herkömmlicher Physiotherapie eine Steigerung der Mobilität erzielen?

Forschungsfrage 4: Verbessert sich die subjektive Lebensqualität durch eine Kombinationsbehandlung aus SSE und herkömmlicher Physiotherapie im Vergleich zur Kontrollgruppe mit ausschließlich herkömmlicher Physiotherapie?

Forschungsfrage 5: Reduziert sich die Sturzangst durch eine Kombinationsbehandlung aus SSE und herkömmlicher Physiotherapie im Vergleich zur Kontrollgruppe mit ausschließlich herkömmlicher Physiotherapie?

1. **Zielgrößen**

Physische Parameter

- Schrittlänge (cm)
- Spurbreite (cm, zusätzlich mit Varianz)
- Walk Ratio (Schritte/min)
- Reservekapazität
- Schrittgeschwindigkeit/Ganggeschwindigkeit (m/s) (normal/maximal/Dual-Task)
- Timed „Up & Go“-Test
- Short Physical Performance Battery (SPPB)

Kognitive Parameter

- Alertness (TAP)
- Geteilte Aufmerksamkeit (TAP)
- Kognitive Flexibilität (TAP)
- Arbeitsgedächtnis (TAP)
- Merkspanne (WAIS-IV)

Sturzangst

- Falls Efficacy Scale (FES-I)

Subjektive Lebensqualität

- Fragebogen zur gesundheitsbezogenen Lebensqualität (EuroQuol)

1. **Studiendesign**

Monozentrisch

Die Studie wird ausschließlich in der geriatrischen Abteilung des Klinikum Bremerhaven- Reinkenheide durchgeführt.

Zweiarmige Studie

Die Studie besteht aus einer Interventions- und einer Kontrollgruppe.

Randomisierung

Es besteht eine Blockrandomisierung mit permutierten Blöcken variabler Länge.

Verblindung

Es besteht eine einfache Verblindung. Die Proband:innen wissen welche Behandlung sie erhalten, die Untersucher:innnen wissen dieses nicht.

1. **Studienpopulation (Kollektiv)**

Studienpopulation

Patient:innen in der geriatrischen Komplexbehandlung im KBR

Ein- und Ausschlusskriterien

Einschlusskriterien

- Gehfähigkeit über kurze Strecken (10m) ohne Hilfsmittel in Begleitung
- Einwilligungsfähigkeit liegt vor (MMSE ≥ 22 Pkt.)
- Ausreichende kognitive Fähigkeiten zur Umsetzung des Trainingsprogramms
- Teilnahme an der stationären geriatrischen Frührehabilitation
- Ausreichende Deutsch- oder Englischkenntnisse

Ausschlusskriterien

- Aphasie in einem Ausmaß, dass die Teilnahme an der Studie nicht möglich ist
- Schwere visuelle Einschränkungen
- Hochgradige Presbyakusis
- starke Beeinträchtigung der körperlichen Funktionalität und Einschränkungen von Funktionen der Arme und Beine mit dem Unvermögen einer Gehfähigkeit

Anzahl der Studienteilnehmer:innen: Insgesamt 60 Teilnehmer:innen

- n= 30 in der Interventionsgruppe
- n= 30 in der Kontrollgruppe

Fallzahlplanung

Für das Primäre Outcome, gemessen mit der SPPB, wurde die randomisiert kontrollierte Machbarkeitsstudie zum Effekt von SSE auf Mobilität und Kognition von Sebastião et al. 2018 als Grundlage für eine Poweranalyse herangezogen. Die Zielgruppe bestand aus älteren Erwachsenen mit Multipler Sklerose (MS) mit leichter bis mittelschwerer Beeinträchtigung (N= 26). Das Training fand im häuslichen Umfeld der Probanden statt. Die Interventionsgruppe erhielt ein 12-wöchiges SSE Training, die Kontrollgruppe erhielt ein Programm bestehend aus minimaler Aktivität und Aufmerksamkeitskontrolle.

Die Fallzahlkalkulation basiert auf den Ergebnissen zur Veränderung der Short Physical Performance Battery (SPPB) von Sebastião et al. (2018). Im Mittel veränderte sich der Score für die SPPB von 8.8 ±2.6 auf 9.5±2.1 Punkte in der Interventionsgruppen und von 7.2±3.3 auf 7.1±4 Punkte in der Kontrollgruppe (η2=0.055). Verwendet wurde die Funktion „F-Test, ANOVA: Repeated measures, within-between interaction” der Software G*Power 3.1.9.4. Unter der Annahme einer α Fehler Wahrscheinlichkeit von 5% und einer Power (1-β) von 95%, sowie zwei Gruppen und zwei Messzeitpunkten und η2=0.055 ergab sich eine erforderliche Gesamtstichprobengröße von N = 58.

Da in der stationären geriatrischen Frührehabilitation der Timed „Up & Go“-Test (TUG) zur Objektivierung einer Veränderung der Mobilität am geläufigsten ist, wurde ebenfalls eine Fallzahlkalkulation basierend auf den Ergebnissen zur Veränderung des TUG von Sebastião et al. (2018) durchgeführt. Im Mittel veränderte sich der Score für den TUG von 10.4 ±3.5 auf 9.7±2.7 Punkte in der Interventionsgruppen und von 15.1±8 auf 14±7.3 Punkte in der Kontrollgruppe (η2=0.014). Verwendet wurde die Funktion „F-Test, ANOVA: Repeated measures, within-between interaction” der Software G*Power 3.1.9.4. Unter der Annahme einer α Fehler Wahrscheinlichkeit von 5% und einer Power (1-β) von 95%, sowie zwei Gruppen und zwei Messzeitpunkten und η2=0.014 ergab sich eine erforderliche Gesamtstichprobengröße von N = 54.

Zwar sind die Ergebnisse der Studienteilnehmer:innen mit MS nicht direkt auf das geriatrische Setting übertragbar, jedoch gibt es bislang keine Studien in diesem Setting die zum Vergleich herangezogen werden könnten. Ebenso verhält es sich mit der Durchführungsdauer der Intervention. Im geriatrischen Setting ist diese auf 2 bis 3 Wochen limitiert. Da sich in der Vergleichsstudie kein linearer Zusammenhang zwischen der Dauer der Intervention und den gemessenen Effekten zeigte, lassen sich diese Parameter nicht ins Verhältnis setzen und somit nicht auf die geplante 2-3-wöchige Intervention anwenden.

Da in der Vergleichsstudie ein:e Proband:in die Teilnahme in der Interventionsgruppe im Verlauf abbrach und von der Datenauswertung exkludiert wurde, haben wir diese Abbruchquote ins Verhältnis zu der errechneten erforderlichen Gesamtstichprobe (N= 58) gesetzt und daraus ein erforderliches N von 60 errechnet.

Rekrutierungswege und –maßnahmen:

Während der Aufnahme der Patient:innen in der Klinik zur geriatrischen Frührehabilitation werden diese auf Erfüllen der Ein- bzw. Ausschlusskriterien überprüft und nach Aufklärung über die Studie und Unterzeichnung der Einwilligungserklärung eingeschlossen.

Rekrutierungszeitraum: 18 Monate

Auf Basis der Poweranalyse planen wir ein erforderliches N von 60. Zur Ermittlung des notwendigen Rekrutierungszeitraums zur Realisierung dieser Fallzahl, überprüften wir die Neuaufnahmen in der geriatrischen Klinik auf Ein- und Ausschlusskriterien über einen Monat hinweg. Innerhalb dieses Monats wurden 107 Patient:innen neu aufgenommen. Davon konnten 24 Patient:innen als geeignete Proband:innen verzeichnet werden. Unter Berücksichtigung einer Drop-out-Rate von 20% aus medizinischen oder persönlichen Gründen ergab die Dokumentation schließlich, dass 19 von den insgesamt 107 neuaufgenommenen Patient:innen in die Studie eingeschlossen werden würden. Daraus ergäbe sich folglich ein Rekrutierungszeitraum von 3 bis 4 Monaten. Aufgrund der Coronapandemie entschlossen wir uns einen längeren Zeitraum zu wählen als rechnerisch notwendig ist und setzen den Rekrutierungszeitraum auf 18 Monate an.

1. **Studienablauf (Vorgehen)**

Verfahren zur Aufklärung und Einholung der Einwilligung

Die Proband:innen werden zu Beginn der Studie mündlich sowie mit der beigefügten Teilnehmerinformation über die Studie aufgeklärt. Die Proband:innen sollen ihre Einwilligung mit ihrer Unterschrift in der beigefügten Einwilligungserklärung dokumentieren. Die unterschriebene Einverständniserklärung (siehe Anlage) ist Voraussetzung für die Studie. Es erfolgt keine Vergütung der Teilnehmer:innen.

Erfassung der Zielgrößen prä und post (Untersuchungen, Messungen, Datenerhebung)

- Ganganalyse mittels sensorischen Messungen und Gait Speed Test unter verschiedenen Bedingungen
  - Walk Ratio
  - Schrittlänge
  - Spurbreite
  - Ganggeschwindigkeit (normal/maximal/Dual-Task)
  - Reservekapazität
- Timed “Up & Go”-Test
- Short Physical Performance Battery (SPPB)
  - Balance
  - Gehgeschwindigkeit
  - Five-Chair Rise
- Fragebogen zur Erfassung von Lebensqualität (EuroQuol)
- Fragebogen zur Erfassung von Angst vor Stürzen (FES-I)
- Testbatterie zur Aufmerksamkeitsdiagnostik (TAP) und Digit-Span zur Erfassung der einfachen verbalen Merkfähigkeit (WAIS-IV)

Im Rahmen von zwei Messterminen (prä und post) werden Daten mit der Short Physical Performance Battery als Testinstrument für die körperliche Funktion der unteren Extremität durchgeführt. Darüber hinaus erfolgt eine computergestützte Messung (TAP) von vier Aufmerksamkeitsparametern (einfache Reaktionsverarbeitungsgeschwindigkeit, geteilte Aufmerksamkeitsleistung, kognitive Umstellfähigkeit, Arbeitsgedächtnisleistung) sowie eine verbal abgeleitete Aufgabe zur Erfassung der einfachen Merkspanne (WAIS-IV). Zur Erfassung der Mobilität werden jeweils 8 Gänge unter 3 Bedingungen (2x normales Gehen, 2x schnelles Gehen, 1x Gehen unter dual-task Bedingung mit jeweils einem Testlauf) mit einem sensorischen Messverfahren (GAITRite oder vergleichbares) sowie der Timed “Up & Go”-Test durchgeführt. Mithilfe von Fragebögen werden die Lebenszufriedenheit (EuroQuol) und die Sturzangst (FES-I) erfasst. Erforderliche sozio-demographische Daten zur Auswertung der erhobenen Parameter werden aus der Patientenakte übernommen, ebenso Haupt- und Nebendiagnosen, Medikamente sowie das geriatrische Assessment (z.B. Timed „Up & Go“-Test und MMST).

Tabelle 1 Datenerhebung

| **Nr.** | **Parameter** | **Messzeitpunkt**  **Beginn (B),**  **Ende (E)** | **Standard im Klinikum (S) /**  **Zusatz (Z)** | **Test** |
| --- | --- | --- | --- | --- |
| 1 | Charakteristika der Teilnehmer (z.B. soziodemographische Daten, Medikamente, Diagnose, geriatrisches Assessment) | B | S | Patientenakte |
| 2 | Gangparameter | B,E | Z, S | GAITRite, Ganggeschwindig-keit (10m) |
| 3 | Physische Parameter | B, E | Z, S | Short Physical Performance Battery  Timed „Up & Go“-Test |
| 4 | Lebensqualität | B, E | Z | EuroQuol |
| 5 | Sturzangst | B, E | Z | FES-I |
| 6 | Kognitive Einschränkungen | B | S | MMST |
| 7 | Aufmerksamkeits- und Exekutivfunktionen | B, E | Z | TAP |
| 8 | Unmittelbare Merkspanne und Arbeitsgedächtnis | B, E | Z | TAP, WAIS-IV |

Randomisierung

Für die Blockrandomisierung mit permutierten Blöcken variabler Länge wurden 12 Blöcke in randomisierter Abfolge mit gleicher Verteilung in vierer und sechser Blöcke erstellt. Dazu wurde in Excel die Funktion „Zufallsbereich“ gewählt. Für die einzelnen Blöcke wurden mit der entsprechenden Funktion jeweils Zufallszahlen für die Probanden ID erstellt. Alle generierten Zufallszahlen, die unter 0.051 lagen, wurden der Gruppe A, alle Zufallszahlen ab 0.051, wurden der Gruppe B zugeordnet. Die Generierung der Zufallszahlen erfolgte solange, bis beide Gruppen innerhalb des 4er und 6er Blocks jeweils gleich häufig vertreten waren. Das Verhältnis zur Gruppenzuteilung beträgt 1:1. Daraus ergibt sich eine Tabelle, der die Proband:innen der Reihenfolge der Studienteilnahme nach zugeordnet werden, wodurch sich eine Zuteilung zur Kontroll- oder Interventionsgruppe ergibt. Nur die Physiotherapeuten erhalten die Kenntnis über die Zuordnung zu den 2 Gruppen durch Einsicht in die erstellte Liste. Somit erfolgt durch die Umsetzung der Blockrandomisierung eine zufällige Zuteilung der Proband:innen zu einer Gruppe, ohne dass eine Manipulation der Zuordnung durch eine, an der Studie beteiligte Person, möglich ist.

Zeitlicher Ablauf (Termine) und Studiendauer für die Proband:innen/ Patient:innen (Flow Chart)

Am Aufnahmetag erfolgt eine ärztliche Überprüfung auf Ein- und Ausschlusskriterien, eine Durchführung des Mini Mental Status Test durch geschultes Personal sowie die Einholung der Einwilligungserklärung zur Teilnahme und Datenschutz.

Alle weiteren kognitiven Tests und die Fragebögen zur Sturzangst und Lebenszufriedenheit werden spätestens am dritten Tag und nach Möglichkeit in einer Sitzung mit einer ungefähren Dauer von 60 Minuten zu Beginn und erneut zum Ende der Studienteilnahme durchgeführt. Sollten Teilnehmer:innen aus gesundheitlichen oder persönlichen Gründen nicht in der Lage sein, kognitive Tests und Fragebögen in einer Sitzung durchzuführen, kann die Durchführung in zwei Termine aufgeteilt werden. Dabei wird die kognitive Testung stets zusammenhängend durchgeführt, um vergleichbare Bedingungen für die Proband:innen zu schaffen.

Die Durchführung der Short Physical Performance Battery (SPPB) sowie die Erhebung der Gangparameter und die Durchführung des Timed “Up & Go”-Test beansprucht in etwa 30 Minuten. Diese erfolgt zusammenhängend und spätestens am dritten Tag nach der Aufnahme und als Re-Testung zum Ende der Studienteilnahme.

Daraus ergibt sich folgender zeitlicher Ablauf

- Aufnahmetag: Überprüfung auf Ein- und Ausschlusskriterien, Durchführung des Mini Mental Status Test, Aufklärung und Einwilligung
- Bis zum dritten Tag des Aufenthaltes: 60 Minuten kognitive Testung inklusive Ausfüllen von Fragebögen und 30 Minuten Funktions- und Mobilitätsmessung
- Über die Zeitdauer der geriatrischen Früh-Rehabilitation „Usual Care“ physiotherapeutische Behandlung in der Kontrollgruppe bzw. Teilnahme an der SSE-Intervention als Kombination aus SSE und „Usual care“ in der Interventionsgruppe
- Re-Testung: 60 Minuten kognitive Testung inklusive Ausfüllen von Fragebögen und 30 Minuten Funktions- und Mobilitätsmessung

Intervention:

Beide Gruppen bekommen die gleiche Anzahl und Zeit in den Trainingssessions insgesamt zur Vergleichbarkeit.

Die Kontrollgruppe bekommt 5x die Woche „Routine Physiotherapie“ (usual care) ca. über 30 Min pro Session.

Die Interventionsgruppe bekommt 2x/ Woche die „Routine Physiotherapie“ und dreimal die Woche für 30 Min das SEE Training. Hierbei wechseln sich das SSE-Training und die „Routine Physiotherapie“ jeweils ab. Dies wird über die gesamte Zeit der geriatrischen Früh-Rehabilitation durchgeführt und so kann je nach Aufenthaltsdauer die Anzahl der Einheiten abweichen, das Verhältnis zur „Routine Physiotherapie bleibt jedoch bestehen. Im SEE Training wird mit einer ansteigenden Trainingsbelastung gearbeitet nach einem strukturierten Trainingsplan. Zu Beginn der Einheit werden die Proband:innen mit dem Teppich vertraut gemacht und erlernen das Gehen über den Teppich. Im Folgenden werden Schrittmuster aus dem Basisniveau erlernt und entsprechend den individuellen Fähigkeiten diese gesteigert. Es werden aus jeder Schwierigkeitskategorie Schrittmuster zur Verfügung gestellt, die dann individuell ausgewählt werden können. Wichtig hierbei ist die Steigerung nach erfolgreichem Erlernen eines Musters.

Die Schrittmuster und Einteilung in Anforderungsniveaus wurden der ursprünglichen Shigematsu Studie (2006 &2008) entnommen und im Rahmen unserer Forschungsgruppe wurde eine Auswahl der zu verwendenden Muster getroffen.

Nach der Gewöhnung an das Trainingsprogramm und dem sicheren Erlernen eines Musters wird eine Dual Task Aufgabe hinzugenommen. Hierbei werden die Proband:innen aufgefordert eine zusätzliche kognitive Aufgabe zu bewältigen während sie das erlernte Muster laufen.

Beispiele für die Anforderungsniveaus der Schrittmuster:

Basisniveau:


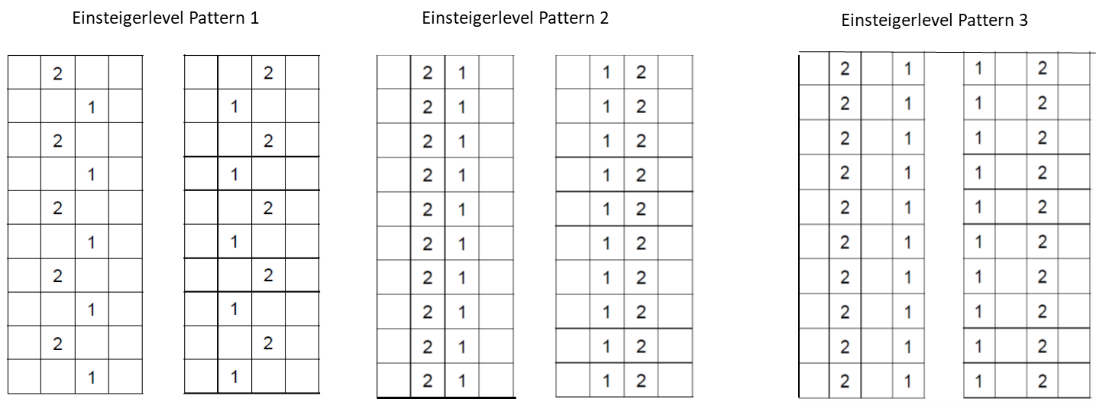

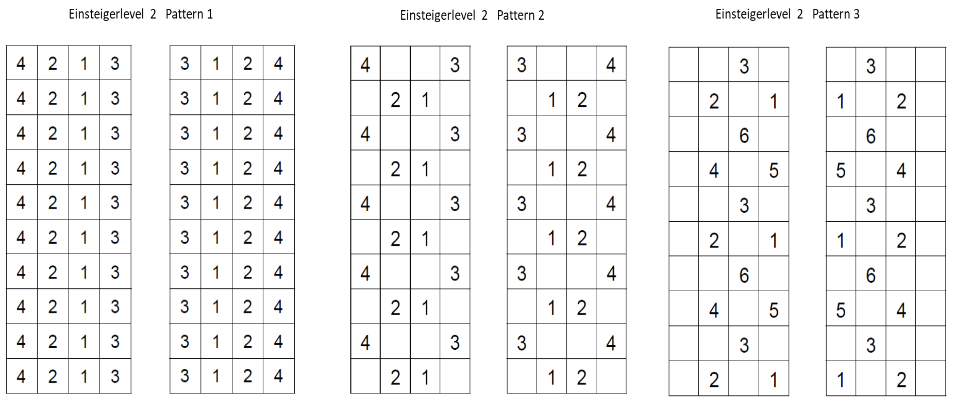


Gesamtdauer der Studie

Für die Proband:innen beträgt die Studiendauer insgesamt zwei bis drei Wochen. Die Studie wird für eine Dauer von 18 Monaten geplant.

1. **Nutzen-Risiko-Abwägung (Sicherheitsaspekte)**

Alle studienbedingten Risiken

Grundsätzliche Risiken liegen in den folgenden Arbeitsbereichen vor:

- die Funktionstests (leichte Muskelverletzungen z.B. blaue Flecken, Zerrungen)
- Geschwindigkeitsmessungen (GAITRite-Sytem oder Gait Speed Test) (Stürze)

Die angewendeten Methoden sind standardisiert und in der Praxis etabliert. Die Teilnehmer:innen werden in der Teilnehmerinformation über die bestehenden Risiken aufgeklärt. Ganggeschwindigkeitsmessungen und Funktionstests werden von entsprechend ausgebildetem, fachkundigen Personal unter gewissenhafter Einhaltung der Sicherheitsstandards und Testprotokolle durchgeführt, so dass die grundsätzlichen Risiken gering sind. Bei der Rekrutierung erfolgt selbstverständlich eine strenge Beachtung der Ausschlusskriterien, so dass eine Überforderung der Teilnehmer:innen kaum zu erwarten ist.

Im Trainingsprogramm kann es in beiden Gruppen (Intervention und Kontrollgruppe) zu leichtem Muskelkater kommen. Allerdings sind die Trainer in beiden Gruppen ausgebildete Physiotherapeuten, die im Klinikalltag die Rehaprogramme für die geriatrischen Patienten leiten und damit über eine langjährige Erfahrung in der Mobilisation von geriatrischen Patienten haben.

Mit der Studie verbundener Nutzen

Bislang findet SSE keine Anwendung in der stationären geriatrischen Frührehabilitation. Die Studie soll den Effekt eines SSE-Trainings im Vergleich zur „Usual care“ physiotherapeutischen Behandlung auf Gangparameter, Exekutivfunktionen, Sturzangst und subjektive Lebens-zufriedenheit untersuchen.

Der mit der Studie verbundene Nutzen besteht in einem wissenschaftlichen Nachweis über ein physiotherapeutisches Trainingsprogramm. Der wissenschaftliche Nachweis darüber, dass ein größerer Effekt in den genannten Funktionsbereichen erzielt werden kann, wenn körperliche und kognitive Fähigkeiten gleichzeitig trainiert werden, kann zu einem besseren Outcome für die Patienten in stationärer geriatrischer Frührehabilitation führen. Die Implementierung von SSE in dieses Setting ist als ökonomisch zu betrachten, da SSE mit geringem zeitlichen und finanziellen Aufwand umgesetzt werden kann und auch im häuslichen Umfeld wetterunabhängig und mit geringer Verletzungsgefahr von den Patienten fortgeführt werden kann.

Der angestrebte wissenschaftliche Erkenntnisgewinn könnte zur Implementierung neuer Trainingsformen in der geriatrischen Frührehabilitation genutzt werden und zu einer Weiterentwicklung dieses Settings beitragen.

Abbruchkriterien (für den individuellen Teilnehmer bzw. für die gesamte Studie)

Die Untersuchungen werden auf Wunsch des Patienten, unabhängig aus welchem Grund, abgebrochen. Eine für den Testleiter sichtbare Ermüdung oder Erschöpfung des Patienten führt ebenfalls zum Abbruch der Untersuchung.

1. **Datenanalyse**

Zunächst werden die erhobenen Daten deskriptiv aufgearbeitet. In einem zweiten Schritt werden die Daten zur Beantwortung der Forschungsfragen mit interferenzstatistischen Verfahren analysiert.

Für die statistische Analyse wird SPSS verwendet. Mithilfe einer Mixed ANOVA (Gruppe x Zeit) wollen wir Effekte auf körperliche Funktion der unteren Extremität, Exekutivfunktionen, Mobilität, gesundheitsbezogene Lebensqualität und Sturzangst untersuchen.

Post-hoc-Analysen mittels t-test dienen der Kontrolle der Gruppen untereinander, um signifikante Effekte von Subtests aufzudecken. Gegebenenfalls werden Korrelationsanalysen (Pearson/Spearman) durchgeführt, um Zusammenhänge zwischen den physischen Funktionstests, der kognitiven Leistungsfähigkeit und den Fragebögen zur gesundheitsbezogenen Lebensqualität und der Sturzangst in verschiedene Richtungen zu ermitteln. Das Signifikanzniveau wird auf α= 5% festgelegt.

**12. Datenmanagement und Datenschutz**

Datenerfassung, -speicherung

Die Daten werden pseudonomisiert verarbeitet. Die Daten werden im Klinikum Bremerhaven Reinkenheide gGmbH unter standardisierten Qualitäts-und Sicherheitsbedingungen aufbewahrt. Zugang zu den Daten haben nur die unmittelbaren Mitarbeiter des Projektes.

Die Daten werden in Papierform und in digitaler Form erfasst und für mindestens 10 Jahre aufbewahrt. Nach Studienende oder -abbruch werden die Dokumente im Archiv des Klinikums gelagert.

Datenweitergabe

Es ist keine Datenweitergabe erforderlich. Alle Daten werden klinikintern ausgewertet und aufbereitet.

Siehe auch beigefügte Datenschutzrechtliche Erklärung mit Widerruf und Datenlöschung.

**13. Umgang mit Biomaterialien: Nicht zutreffend**

**14. Probandenversicherung (sofern zutreffend): Nein**

**Literatur**

Al-Yahya, E., Dawes, H., Smith, L., Dennis, A., Howells, K., & Cockburn, J. (2011). Cognitive motor interference while walking: a systematic review and meta-analysis. *Neuroscience & Biobehavioral Reviews*, *35*(3), 715-728.

Ball, K., Berch, D. B., Helmers, K. F., Jobe, J. B., Leveck, M. D., Marsiske, M., & ACTIVE Study Group. (2002). Effects of cognitive training interventions with older adults: a randomized controlled trial. *Jama*, *288*(18), 2271-2281.

Bishnoi, A., Holtzer, R., & Hernandez, M. E. (2021). Brain Activation Changes While Walking in Adults with and without Neurological Disease: Systematic Review and Meta-Analysis of Functional Near-Infrared Spectroscopy Studies. *Brain sciences*, *11*(3), 291.

Brand, M., & Markowitsch, H. J. (2004). Frontalhirn und Gedächtnis im Alter. *NeuroGeriatrie*, *1*, 1-11.

Cabeza, R., Anderson, N. D., Locantore, J. K., & McIntosh, A. R. (2002). Aging gracefully: compensatory brain activity in high-performing older adults. *Neuroimage*, *17*(3), 1394-1402.

Cabeza, R., Daselaar, S. M., Dolcos, F., Prince, S. E., Budde, M., & Nyberg, L. (2004). Task-independent and task-specific age effects on brain activity during working memory, visual attention and episodic retrieval. *Cerebral cortex*, *14*(4), 364-375.

Cohen, J. A., Verghese, J., & Zwerling, J. L. (2016). Cognition and gait in older people. *Maturitas*, *93*, 73-77.

Colcombe, S., & Kramer, A. F. (2003). Fitness effects on the cognitive function of older adults: a meta-analytic study. *Psychological science*, *14*(2), 125-130.

Falck, R. S., Davis, J. C., Best, J. R., Crockett, R. A., & Liu-Ambrose, T. (2019). Impact of exercise training on physical and cognitive function among older adults: a systematic review and meta-analysis. *Neurobiology of aging*, *79*, 119-130.

Falck, R. S., Landry, G. J., Best, J. R., Davis, J. C., Chiu, B. K., & Liu-Ambrose, T. (2017). Cross-sectional relationships of physical activity and sedentary behavior with cognitive function in older adults with probable mild cognitive impairment. *Physical therapy*, *97*(10), 975-984.

Fisseha, B., Janakiraman, B., Yitayeh, A., & Ravichandran, H. (2017). Effect of square stepping exercise for older adults to prevent fall and injury related to fall: systematic review and meta-analysis of current evidences. *Journal of exercise rehabilitation*, *13*(1), 23.

Hausdorff, J. M., Yogev, G., Springer, S., Simon, E. S., & Giladi, N. (2005). Walking is more like catching than tapping: gait in the elderly as a complex cognitive task. *Experimental brain research*, *164*(4), 541-548.

Hedden, T., & Yoon, C. (2006). Individual differences in executive processing predict susceptibility to interference in verbal working memory. *Neuropsychology*, *20*(5), 511.

Holtzer, R., Ross, D., & Izzetoglu, M. (2020). Intraindividual variability in neural activity in the prefrontal cortex during active walking in older adults. *Psychology and Aging*, *35*(8), 1201.

Holtzer, R., Verghese, J., Xue, X., & Lipton, R. B. (2006). Cognitive processes related to gait velocity: results from the Einstein Aging Study. *Neuropsychology*, *20*(2), 215.

Jadczak, A. D., Makwana, N., Luscombe-Marsh, N., Visvanathan, R., & Schultz, T. J. (2018). Effectiveness of exercise interventions on physical function in community-dwelling frail older people: an umbrella review of systematic reviews. *JBI Evidence Synthesis*, *16*(3), 752-775.

Kearney, F. C., Harwood, R. H., Gladman, J. R., Lincoln, N., & Masud, T. (2013). The relationship between executive function and falls and gait abnormalities in older adults: a systematic review. *Dementia and geriatric cognitive disorders*, *36*(1-2), 20-35.

Logan, J. M., Sanders, A. L., Snyder, A. Z., Morris, J. C., & Buckner, R. L. (2002). Under-recruitment and nonselective recruitment: dissociable neural mechanisms associated with aging. *Neuron*, *33*(5), 827-840.

Lundin-Olsson, L., Nyberg, L., & Gustafson, Y. (1997). Stops walking when talking as a predictor of falls in elderly people. *Lancet*, *349*(9052), 617.

Montero‐Odasso, M., Verghese, J., Beauchet, O., & Hausdorff, J. M. (2012). Gait and cognition: a complementary approach to understanding brain function and the risk of falling. *Journal of the American Geriatrics Society*, *60*(11), 2127-2136.

Morrison, J. H., & Baxter, M. G. (2012). The ageing cortical synapse: hallmarks and implications for cognitive decline. *Nature Reviews Neuroscience*, *13*(4), 240-250.

Oswald, W. D., Gunzelmann, T., Rupprecht, R., & Hagen, B. (2006). Differential effects of single versus combined cognitive and physical training with older adults: the SimA study in a 5-year perspective. *European journal of ageing*, *3*(4), 179.

Reuter-Lorenz, P. A., Jonides, J., Smith, E. E., Hartley, A., Miller, A., Marshuetz, C., & Koeppe, R. A. (2000). Age differences in the frontal lateralization of verbal and spatial working memory revealed by PET. *Journal of cognitive neuroscience*, *12*(1), 174-187.

Sánchez-Arenas, R., Doubova, S. V., Bernabe-Garcia, M., Gregory, M. A., Mejía-Alonso, L. A., Orihuela-Rodríguez, O., & Shigematsu, R. (2020). Double-task exercise programmes to strengthen cognitive and vascular health in older adults at risk of cognitive decline: protocol for a randomised clinical trial. *BMJ open*, *10*(12), e039723.

Sebastião, E., McAuley, E., Shigematsu, R., Adamson, B. C., Bollaert, R. E., & Motl, R. W. (2018). Home-based, square-stepping exercise program among older adults with multiple sclerosis: results of a feasibility randomized controlled study. *Contemporary clinical trials*, *73*, 136-144.

Shellington, E. M., Reichert, S. M., Heath, M., Gill, D. P., Shigematsu, R., & Petrella, R. J. (2018). Results from a feasibility study of square-stepping exercise in older adults with type 2 diabetes and self-reported cognitive complaints to improve global cognitive functioning. *Canadian journal of diabetes*, *42*(6), 603-612.

Shigematsu, R., & Okura, T. (2006). A novel exercise for improving lower-extremity functional fitness in the elderly. *Aging clinical and experimental research*, *18*(3), 242-248.

Shigematsu, R., Okura, T., Nakagaichi, M., Tanaka, K., Sakai, T., Kitazumi, S., & Rantanen, T. (2008). Square-stepping exercise and fall risk factors in older adults: a single-blind, randomized controlled trial. *The Journals of Gerontology Series A: Biological Sciences and Medical Sciences*, *63*(1), 76-82.

Shigematsu, R., Okura, T., Sakai, T., & Rantanen, T. (2008b). Square-stepping exercise versus strength and balance training for fall risk factors. *Aging clinical and experimental research*, *20*(1), 19-24.

Shigematsu, R., Okura, T., Nakagaichi, M., & Nakata, Y. (2014). Effects of exercise program requiring attention, memory and imitation on cognitive function in elderly persons: a non-randomized pilot study. *J Gerontol Geriatric Res*, *3*(02), 147.

Smith, E., Cusack, T., & Blake, C. (2016). The effect of a dual task on gait speed in community dwelling older adults: A systematic review and meta-analysis. *Gait & posture*, *44*, 250-258.

Soshi, T., Andersson, M., Kawagoe, T., Nishiguchi, S., Yamada, M., Otsuka, Y., ... & Sekiyama, K. (2021). Prefrontal Plasticity after a 3-Month Exercise Intervention in Older Adults Relates to Enhanced Cognitive Performance. *Cerebral Cortex*.

Szameitat, A. J., Schubert, T., Müller, K., & Von Cramon, D. Y. (2002). Localization of executive functions in dual-task performance with fMRI. *Journal of cognitive neuroscience*, *14*(8), 1184-1199.

Swoboda, W., Sieber, C. Rehabilitation in der Geriatrie. *Internist* 51, 1254-1261 (2010).

Teixeira, C. V. L., Gobbi, S., Pereira, J. R., Vital, T. M., Hernandéz, S. S. S., Shigematsu, R., & Gobbi, L. T. B. (2013). Effects of square‐stepping exercise on cognitive functions of older people. *Psychogeriatrics*, *13*(3), 148-156.

Webber, S. C., Porter, M. M., & Menec, V. H. (2010). Mobility in older adults: a comprehensive framework. *The gerontologist*, *50*(4), 443-450.

Willis, S. L., Tennstedt, S. L., Marsiske, M., Ball, K., Elias, J., Koepke, K. M., & ACTIVE Study Group, F. T. (2006). Long-term effects of cognitive training on everyday functional outcomes in older adults. *Jama*, *296*(23), 2805-2814.

Woollacott, M., & Shumway-Cook, A. (2002). Attention and the control of posture and gait: a review of an emerging area of research. *Gait & posture*, *16*(1), 1-14.

World Health Organization. (2015). *World report on ageing and health*. World Health Organization.

Yeom, H. A., Fleury, J., & Keller, C. (2008). Risk factors for mobility limitation in community-dwelling older adults: a social ecological perspective. *Geriatric nursing*, *29*(2), 133-140.
