# Supplementary material for: Effects of Square-Stepping Exercise on cognitive function in early geriatric rehabilitation: A randomized controlled explorative study
Source: PLoS One. 2026 Jan 5;21(1):e0338695. doi: 10.1371/journal.pone.0338695 (PMC12768348; doi:10.1371/journal.pone.0338695)
Supplement: Attachment — Submitted filename: Reviewers comments PLOS.docx [file pone.0338695.s005.docx]

Response to reviewer #1: Thank you for taking the time to review our manuscript. We appreciate your constructive comments, which have helped us to improve the clarity and overall quality of the paper.

1. Overall an interesting manuscript. More major concerns involved the data being collected in 2021-2022 but now being published in 2025 - an explanation for the delay and decision to publish now may be helpful. The 40 references are also all from 2023 or before and the majority were published pre 2020 and therefore pre COVID. It would be helpful to update some of the references to make the manuscript as relevant to the present as possible.

**Response:** Thank you for your valuable feedback, and the careful reading. We would like to clarify that although data collection for the study was conducted between 2021 and 2022, initial findings were already published in April 2024. This prior publication is referenced in the manuscript, e.g. in line 203.

The current manuscript was first submitted to PLOS in March 2025. Based on our experience, this timeline reflects a typical duration for data analysis, interpretation, and manuscript preparation within a research group.

We have revised the reference list and added the following citations to further support the findings of the manuscript:

- Siqueira VAAA, Shigematsu R, Sebastião E. Stepping towards health: a scoping review of square-stepping exercise protocols and outcomes in older adults. BMC Geriatr. 2024;24; 590. doi: 10.1186/s12877-024-05187-8
- Franco-García JM, Pérez-Gómez J, Castillo-Paredes A, Redondo PC, Rojo-Ramos J, Mayordomo-Pinilla N, Villafaina S, Gómez-Álvaro MC, Melo-Alonso M, Carlos-Vivas J. Effects of Twelve Weeks of Square Stepping Exercises on Physical and Cognitive Function and Plasma Content of SMP30: A Randomised Control Trial. Geriatr. 2025; 10: 22. doi:10.3390/geriatrics10010022

2. The text describes how. 72 patients were included from 307 patients and Figure 1 indicates that 192 were excluded due to not meeting inclusion criteria. However, it would be helpful to understand which criteria the patients failed if this data is available. If nearly 2/3 of patients were excluded for not meeting the inclusion criteria then greater detail is required to ensure that there was not a selection bias hidden within these exclusions. It would also be helpful if the authors provided some examples of the 18 'other reasons' why patients were not included for randomisation.

**Response:** From the beginning, it was evident that the SSE training program was not applicable to all patients due to their varied diagnoses. The most common reason patients were unable to participate was that they could not walk ten meters independently. This is due to the diverse population of patients in the early stages of geriatric rehabilitation. This population consists of patients with acute neurological and orthopedic conditions. Due to paresis after stroke and fractures with partial weight-bearing, meeting this inclusion criterion was impossible. Because of this heterogeneity, the functional levels of patients in early geriatric rehabilitation vary. The SSE is intended for approximately one-third of patients with a higher functional level. Unfortunately, it is impossible to create a detailed analysis based on the data.

3. Several small language and grammar errors were noted and corrections suggested below:

Line 218 'executive function such as'

**Response:** The word 'such' was added (L219)

Line 236 'we expected a positive/negative effect on cognitive function'

**Response:** The word 'positive' was added (L 238)

Line 259 sentence needs to be revised as 'excluded were inpatients with aphasia to an extent that participation in the study is not possible' is incorrect use of tenses and unclear.

**Response:** The wording has been replaced by 'Inpatients with severe aphasia, …, were excluded' (LL263 – 266).

Line 314 to 321 - alertness subtest and divided attention tests are not explained clearly. Consider expanding these explanations or clarifying the language as the current wording is not reader friendly for those who are not already aware of these tests.

**Response:** The description has been revised in LL321-331 and made more detailed.

Line 339 - 'higher scores indicate higher levels of concerns about falling' OR 'higher scores indicate a higher level of concern about falling'

**Response:** The wording has been replaced by 'higher scores indicate a higher level of concern about falling' (L 348).

426 'effects on cognition'

**Response:** We have changed accordingly to ‘cognition’ (L 430).

428 'further research on this specific area? Field?...'

**Response:** Thank your pointing out the missing clarity we have now changed to:

‘As the number of studies on the effect of SSE on cognitive function in older people is still limited, further research in this specific research field with a larger study sample and a longer intervention period is required’ (LL 455-458).

Response to reviewer #2: We are grateful for your careful review and thoughtful suggestions. Your feedback has been helpful in refining the manuscript and addressing important points.

Reviewer #2: This paper reports on the secondary endpoint measures of cognitive function from a randomized trial of conventional physiotherapy (cPT) vs CPT plus Square-Stepping Exercise (SSE) in patients undergoing inpatient early geriatric rehabilitation.

Several aspects of this study limit one's ability to draw meaningful conclusions. In particular:

1. The sample size is quite small. It is said to have been derived for testing the primary endpoint (Short Physical Performance Battery), but no details of that calculation or the underlying assumptions are provided. To fully interpret the results in this present manuscript, it would be important to know what a clinically meaningful difference would be for the secondary endpoints reported here and what power this sample size would have yielded to observe those differences. The statistical analysis appears to have been carried out appropriately in a technical sense, but some conclusions may be overstated.

**Response:** We also discussed this point in detail in our study group. A reference to the detailed calculation of the sample size in the previous publication can be found in LL 468-471. The results were not reported as they were not part of the statistical analysis of this manuscript. However, they are freely accessible in the cited reference [26].

To the best of our knowledge, there is no established value for a clinically meaningful difference, apart from a statistically significant result, for FES-I (e.g. [https://doi.org/10.1093/ageing/afad055](NULL)) and TAP. Since the VAS was used in EuroQol, no clinically meaningful difference can be derived here.

2. The short duration of the intervention (3 weeks) and the short training duration (30 minutes per session) may simply be too little to have an impact on cognitive function.

**Response:** We also saw this point as a limiting factor, as shown in L 478. To clarify, the following was added: ‘However, in the specific setting of early geriatric rehabilitation, the duration of intervention is limited to three weeks’ (LL483-484).

3. The preliminary disclosure pages provide inconsistent information regarding the availability of the data. The response to the data availability section states "Yes - all data are fully available without restriction" but later in that section, the authors indicate that the data cannot be made publicly available and are available for "researchers who meet the criteria..."

**Response:** We are very sorry for the confusion. This must be an error in the submission process. As described in the manuscript: The data that support the findings of this study are available on reasonable request from the corresponding author.

Unfortunately, it is not possible to provide all the data in full because this is a clinical sample taken during normal hospital operations. Some of the collected data comes from patients' medical records. Patients did not agree to make their data available in a repository when they signed the consent form to participate in the study.

4. The manuscript needs careful proofreading. For example, lines 230-231 are not a sentence. (It appears that the word 'as' should be deleted.) In line 428 "lager" should be "larger".

**Response:** We changed this accordingly in L 456. The manuscript has been proofread thoroughly.
