# Supplementary material for: Effects of Square-Stepping Exercise on cognitive function in early geriatric rehabilitation: A randomized controlled explorative study
Source: PLoS One. 2026 Jan 5;21(1):e0338695. doi: 10.1371/journal.pone.0338695 (PMC12768348; doi:10.1371/journal.pone.0338695)
Supplement: Attachment — Submitted filename: Final Answer Reviewers Comments.docx [file pone.0338695.s006.docx]

Reviewers' comments:

Reviewer's Responses to Questions

**Comments to the Author**

1. If the authors have adequately addressed your comments raised in a previous round of review and you feel that this manuscript is now acceptable for publication, you may indicate that here to bypass the “Comments to the Author” section, enter your conflict of interest statement in the “Confidential to Editor” section, and submit your "Accept" recommendation.

Reviewer #2: (No Response)

2. Is the manuscript technically sound, and do the data support the conclusions?

The manuscript must describe a technically sound piece of scientific research with data that supports the conclusions. Experiments must have been conducted rigorously, with appropriate controls, replication, and sample sizes. The conclusions must be drawn appropriately based on the data presented.

Reviewer #2: Partly

3. Has the statistical analysis been performed appropriately and rigorously?

Reviewer #2: Yes

4. Have the authors made all data underlying the findings in their manuscript fully available?

The [PLOS Data policy](http://?) requires authors to make all data underlying the findings described in their manuscript fully available without restriction, with rare exception (please refer to the Data Availability Statement in the manuscript PDF file). The data should be provided as part of the manuscript or its supporting information, or deposited to a public repository. For example, in addition to summary statistics, the data points behind means, medians and variance measures should be available. If there are restrictions on publicly sharing data—e.g. participant privacy or use of data from a third party—those must be specified.

Reviewer #2: No

**Answer**

Statement on data availability: German data safety law prohibits the general provision of all collected data. Nevertheless, the data can be made available upon reasonable request from the corresponding author. This has already been declared in the manuscript. With due respect we acknowledge that research data should be made available but German data law prohibits an “unrestricted” public availability. In addition, as this was requested in the ethical clearance that only upon request data will be made available. Therefore, for future researchers they can always ask for the data to be incorporated into their research, this will be no problem.

5. Is the manuscript presented in an intelligible fashion and written in standard English?

PLOS ONE does not copyedit accepted manuscripts, so the language in submitted articles must be clear, correct, and unambiguous. Any typographical or grammatical errors should be corrected at revision, so please note any specific errors here.

Reviewer #2: Yes

6. Review Comments to the Author

Please use the space provided to explain your answers to the questions above. You may also include additional comments for the author, including concerns about dual publication, research ethics, or publication ethics. (Please upload your review as an attachment if it exceeds 20,000 characters)

Reviewer #2: While the statistical analysis is technically correct, I remain concerned that there is too much potential for selection bias in the small subset of patients included in the study, a lack of sufficient information to draw conclusions about these secondary endpoints from the small (and potentially biased) sample, and the limitations of the intervention and training duration. Combined, these factors make it difficult to reliably interpret the material as presented and thus limit the contribution of this manuscript to the field.

**Answer to Reviewers Comment :**

We fully understand these concerns and agree with some of them.

The sample size is indeed small, so generalization is not possible. Nevertheless, this is one of the first studies in the field of early geriatric rehabilitation. The study's framework conditions are determined by this setting. It is not comparable to a community-dwelling setting. The public health system defines the intervention period, which is always the same. Although it is shorter than that for community-dwelling individuals, it is defined by law. Since the implementation duration is a general law, this study is comparable to others in the field of geriatrics with the same general law in terms of implementation duration (e.g. Austria).

We acknowledge that the study included a small subset of patients. However, with all due respect, we would like to point out that we collected detailed baseline characteristics (e.g., Barthel, SPPB, MMSE) to clarify the functional level of the patients included in the study. Of course, the results cannot be applied to every geriatric ward worldwide because the functional level can vary. Nevertheless, we believe that this basic information on the patients' functional levels can be generalized because we used popular measurements, such as the SPPB, which is used in geriatric research. In order to carry out this type of intervention in this population, a subsample with a higher functional level had to be formed.

Furthermore, it is important to bear in mind that little research has been conducted in this specific area to date. Despite the small sample size, this is still a randomized controlled study.

In summary, we fully understand these concerns. However, the study was conducted within the legal framework for early geriatric rehabilitation in Germany. This means that the results are also transferable to early geriatric rehabilitation programs that are subject to the same framework conditions (e.g., in Germany and Austria). The functional level of the patients in the subsample is made transparent by the baseline characteristics.

To highlight this more clearly, we have added the following to the imitations: "When interpreting the results, it is also important to note that the inclusion criteria resulted in a small subsample of the geriatric population. This gives rise to a potential selection bias. The results are therefore only transferable to a population with a comparable functional level. " in LL 446-449.

7. PLOS authors have the option to publish the peer review history of their article ([what does this mean?](http://?)). If published, this will include your full peer review and any attached files.

If you choose “no”, your identity will remain anonymous but your review may still be made public.

**Do you want your identity to be public for this peer review?** For information about this choice, including consent withdrawal, please see our [Privacy Policy](http://?).

Reviewer #2: No
